# Life cycle assessment of microgreen production: effects of indoor vertical farm management on yield and environmental performance

**DOI:** 10.1038/s41598-023-38325-0

**Published:** 2023-07-13

**Authors:** Michael G. Parkes, Duarte Leal Azevedo, Ana Celeste Cavallo, Tiago Domingos, Ricardo F. M. Teixeira

**Affiliations:** 1 Environment and Technology Centre, LARSyS-Laboratory of Robotics and Engineering System, MARETEC-Marine, Instituto Superior Técnico, Universidade de Lisboa, Av. Rovisco País 1, 1049-001 Lisboa, Portugal; 2 Canguru Foods, Lda, Social Enterprise, Rua José Dias Simão S/N, TAGUSVALLEY – Parque de Ciência e Tecnologia, 2200-062 Abrantes, Portugal; 3 CIRSA - Centro Interdipartimentale Di Ricerca Per Le Scienze Ambientali, Alma Mater Studiorum - University of Bologna, Via Dell’Agricoltura 5, 48123 Ravenna, Italy

**Keywords:** Environmental impact, Environmental health

## Abstract

The global production of plant-based foods is a significant contributor to greenhouse gas emissions. Indoor vertical farms (IVFs) have emerged as a promising approach to urban agriculture. However, their environmental performance is not well understood, particularly in relation to operational choices where global warming potentials (GWP) can vary between 0.01–54 kg CO_2_e/kg^−1^ of leafy greens produced. We conducted a life cycle assessment (LCA) of a building-integrated IVF for microgreen production to analyse a range of operational conditions for cultivation: air temperature, CO_2_ concentration, and photoperiod. We analyzed a dynamic LCA inventory that combined a process-based plant growth model and a mass balance model for air and heat exchange between the chamber and the outside. Results showed that the GWP of IVFs can vary greatly depending on the operation conditions set, ranging from 3.3 to 63.3 kg CO_2_e/kg^−1^. The optimal conditions for minimizing GWP were identified as 20 ℃, maximum CO_2_ concentration in the chamber, and maximum photoperiod, which led to a minimum GWP of 3.3 kg CO_2_e/kg^−1^ and maximum production of 290.5 kg fresh weight week^-1^. Intensification of production thus led to lower impacts because the marginal increase in yield due to increased resource use was larger than the marginal increase in impact. Therefore, adjusting growing conditions is essential for the sustainability of urban food production.

## Introduction

The production and transportation of plant-based food products contribute significantly to greenhouse gas (GHG) emissions, particularly in urban areas^1,2^. Working to reduce emissions by producing food closer to or within urban centres is a potential solution^3^. Urban Agriculture (UA) is a term generically applied to food produced in urban infrastructures in peri-urban areas or cities and requires less transport^4,5^. One form of UA is indoor vertical farms (IVF) which is a soil-less growing system that involves stacked farming structures^6,7^. UA has the potential to decrease downstream emissions by reducing or avoiding packaging and transportation thanks to the physical proximity to consumers^8–10^. However, UA requires significant built infrastructure, agricultural technology (ag-tech), and material inputs such as fertilizers, substrates, seeds, and industrial CO_2_ which contribute to upstream emissions. Compared to conventional farming, UA ag-tech relies on artificial lighting from Light Emitting Diodes (LEDs) and climate systems for heating and cooling, which result in higher emissions per unit of product^5,11,12^. Studies indicate that electricity consumption accounts for up to 93% of all GHG emissions in UA food production using IVF^13,14^. In order to ensure that UA products are produced with minimum possible emissions, IVFs will need to run more efficiently, minimizing the effects of those added emissions in comparison to conventional farming.

Bridging this knowledge gap is crucial for developing strategies to reduce greenhouse gas emissions in UA. This presents a major challenge in UA surrounding environmental performance. Limited transferability of conclusions from individual studies makes it difficult to determine how best to tackle this challenge for new IVFs and UA projects. Confounding variables such as growing conditions, ag-tech and facility types further complicate efforts to compare and generalise individual studies^15,16^. It is critical to address this knowledge gap to fully understand the environmental implications of IVF in UA and to develop strategies to optimize its sustainability.

Life cycle assessment (LCA) is a crucial framework to estimate the environmental performance of various production systems by addressing both direct and indirect impacts across the entire supply chain^5,15,16^. This approach has been widely used to evaluate individual farms and IVFs for food production^13^. However, the reported emissions of these assessments range from 0.01 to 54 kg CO_2_e/kg for leafy greens produced in UA farms^11,17^, due to lack of methodological consistency and intrinsic differences between systems^15,18^. Such variations in the findings of different LCA studies are often attributed to several factors, such as crop type, cultivation methods, yield, ag-tech installed, and operational management of the facility^5,17,19^. However, one critical factor that has not been adequately assessed is the latter, operational management, which plays a crucial role in the global performance of IVFs. Most LCA studies available to date focus on a fixed set of operational conditions for each IVF assessed, which further exacerbates the knowledge gap regarding the effect of operational management on the environmental performance of IVFs^20,21^. Therefore, developing an LCA study that accounts for the impact of changes in plant growth conditions on the environmental performance of IVFs is necessary to evaluate the sustainability of UA and urban food production systems.

A prospective building-integrated IVF technology that aimed to reduce supply chains and GHG emissions of food production was installed to produce a *Brassica oleracea* species as microgreens in the basement of one of seven buildings on a university campus in Lisbon, Portugal^16^. The installation included an IVF and a preparation work area, both equipped with Internet-of-Things (IoT) devices and connected to the building's water and electricity supply. The IVF consists of a 32 m^2^ growth chamber with vertical hydroponics and an LED lighting system, with a proprietary software for production control and monitoring. One past LCA study of this unit, like others^15,18^, applied fixed inputs for the Life Cycle Inventory (LCI) to assess the supply of 1 kg of microgreen and GHG emissions of the technology but not how changes in operational conditions affect the yield and its relationship to environmental impacts.

In this study, we conducted a comprehensive LCA for microgreens grown in this building-integrated IVF in Lisbon by exploring an entire option space of combinations of operational parameters. This approach was taken to address the lack of comprehensive assessment of the effects of operational parameters: air temperature in the chamber, CO_2_ concentration, and photoperiod. A dynamic LCI approach was used to define the option space, which explicitly models physical plant growth depending on inputs, operational parameters, and growing conditions. Furthermore, we used Life cycle impact assessment (LCIA) to assess the LCI for each combination of operational parameters to determine GHG emissions with impacts specific to climate change and indicators for humans, marine, freshwater, and terrestrial ecotoxicity^20,22^. The ultimate goal was to identify the set of conditions that minimize emissions per kg of microgreens produced, inform the most sustainable practices for UA, and management of IVF operations to address the global challenges of climate change and food production.

## Results

**Figure 1:**
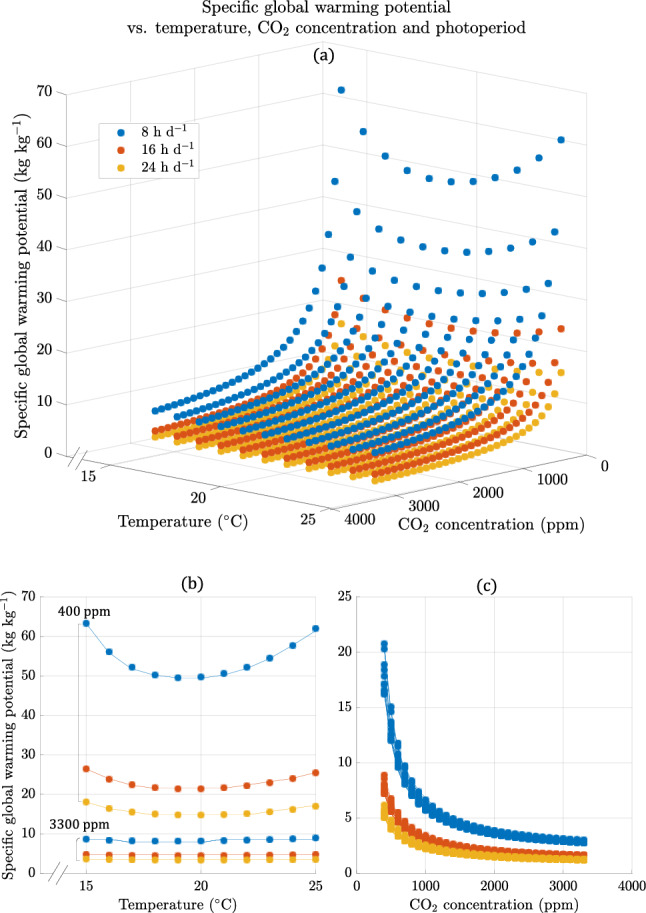
(**a**) Specific GWP as a function of temperature, CO_2_ concentration and photoperiod: a three-dimension representation accompanied by two-dimensional ones as a function of temperature (**b**) and as a function of CO_2_ concentration (**c**). Only three values for photoperiod are represented for clarity: 8 h d^−1^ (blue), 16 h d^−1^ (orange) and 24 h d^−1^ (yellow). In (**b**) and (**c**), points represent temperatures and concentrations values that were explicitly modelled and lines are an interpolation between each value used in the model.

Figure 1 shows the specific Global Warming Potential (GWP), measured in emissions of CO_2_ equivalents per 1 kilogram (kg) unit fresh weight (FW) of kale (*Brassica oleracea, var. acephala*) microgreens for various combinations of air temperatures, photoperiods, and CO_2_ concentrations. The trends observed indicate that increasing the CO_2_ concentration and photoperiod resulted in lower GWP at all temperatures studied. The minimum achievable GWP was 3.34 kg CO_2_e/kg obtained at the maximum CO_2_ concentration (3300 ppm) and photoperiod (PP) 24 h d^−1^. Maximum GWP was 63.34 kg CO_2_e/kg observed at the lowest CO_2_ concentration (400 ppm) and photoperiod 8 h d^−1^ for all temperatures considered. These findings suggest that maximizing CO_2_ concentration and photoperiod can minimize GWP, irrespective of temperature.

The results show that a temperature range of 19 to 20 °C minimized specific GWP for all CO_2_ concentrations and photoperiods, as demonstrated in Fig. 1b. Additionally, at a constant temperature of 20 °C and photoperiod of 8 h d^-1^, increasing the dosing of CO_2_ into the IVF had the highest impact on reducing GWP, as shown in Fig. 1c. Increasing CO_2_ concentration by 1000 ppm from 400 to 1400 ppm resulted in a larger reduction in GWP (36.4 kg CO_2_e/kg) than a similar increase from 1400 to 2400 ppm (3.7 kg CO_2_e/kg). However, the marginal decrease in specific GWP with increasing CO_2_ concentration is smaller for larger concentrations.

**Figure 2:**
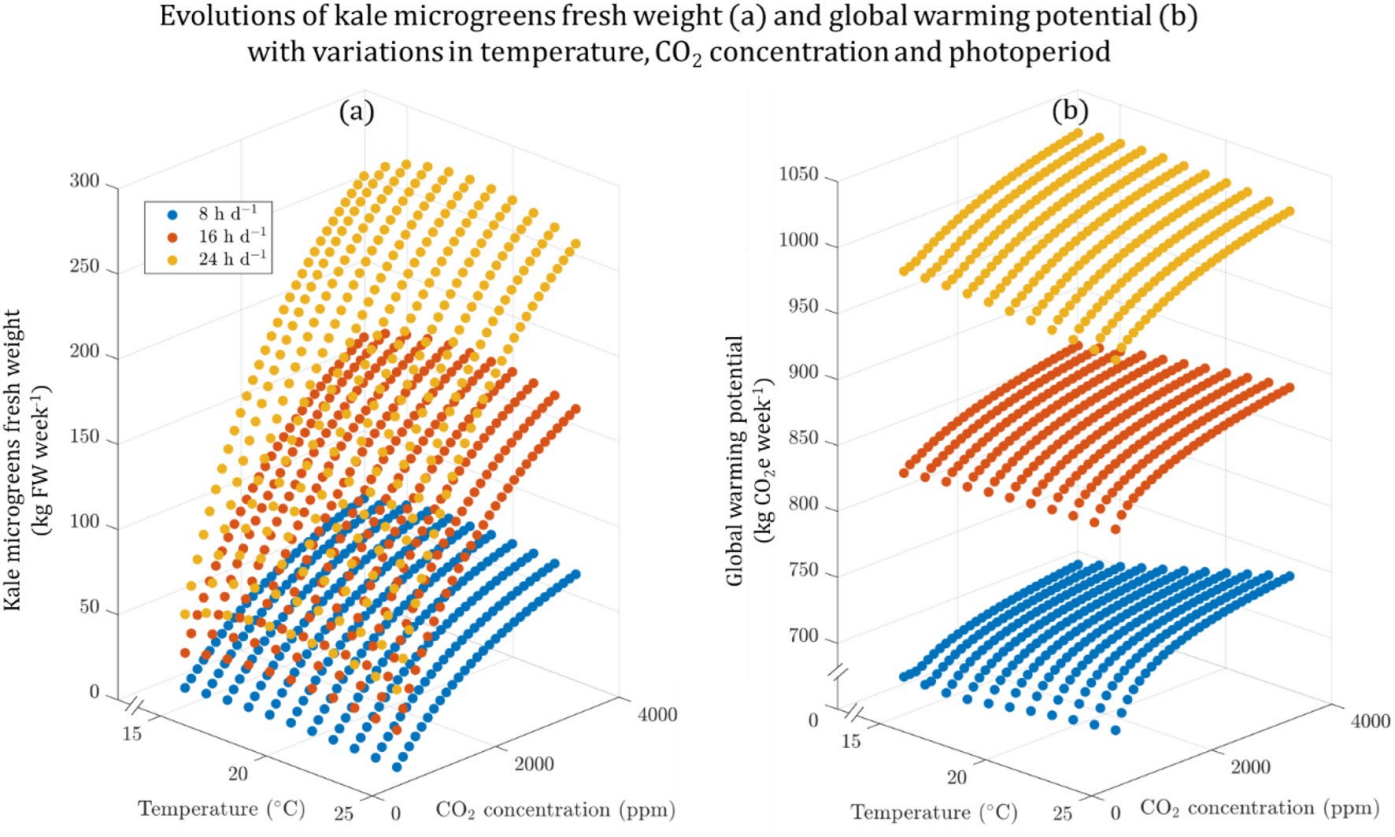
Kale microgreen fresh weight yield (**a**) and absolute GWP (**b**) as a function of temperature, CO_2_ concentration, and photoperiod (only three values represented for clarity: 8 h d^−1^, blue, 16 h d^−1^, orange, and 24 h d^−1^, yellow).

The evolution of specific GWP depended on the weekly kale production. Both absolute GWP (kg CO_2_e/week) and yield increased with photoperiod and CO_2_ concentration (Fig. 2). The absolute GWP at its highest was 978 kg CO_2_e week^−1^ at 17 ℃ and maximum photoperiod and CO_2_ concentration, resulting in a yield of 285.2 kg FW week^-1^. The lowest absolute GWP was 674 kg CO_2_ e week^−1^ at 17 ℃ with minimum photoperiod and CO_2_ concentration, which resulted in a yield of 12.9 kg CO_2_ e week^−1^. The maximum yield of 290.5 kg week^-1^ was achieved at the temperature of 20 ℃. Environmental conditions that minimized GWP per unit weight of kale were the same as that maximized production; the highest CO_2_ concentration (4000 ppm) and photoperiod (PP = 24 h d^−1^). This indicates that by intensifying the operational conditions and increasing resource consumption, the marginal increase in yield outweighed the marginal increase in associated environmental impacts, leading to a decrease in GWP per functional unit.

**Figure 3:**
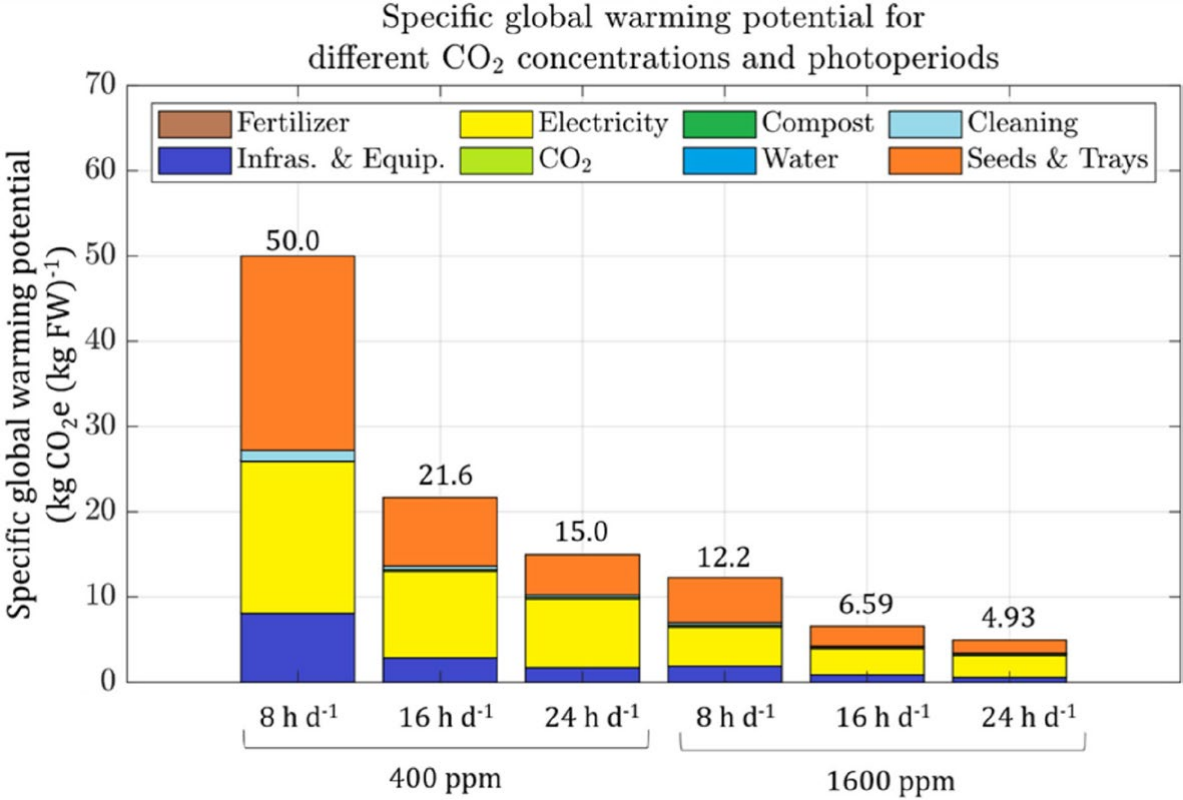
Specific GWP contribution of the different inputs required to produce kale in the plant farm, for two CO_2_ concentrations, 400 and 1600 ppm, and three photoperiods, 8, 16, and 24 h d^−1^, and a temperature of 20 ℃.

The results of the climate change impact assessment showed that electricity use and seeded trays were the two largest contributors to the impact category of GWP, accounting for 35% to 54% and 30% to 46% of the total impact, respectively (Fig. 3). Infrastructure and equipment accounted for 11% to 16% of the total impact and remained constant across all conditions tested. CO_2_ supply from the cylinder was a minor contributor to the impact category, ranging from 0.5% to 4.8%. The specific GWP of electricity supply was 0.35 kg CO_2_e (kWh)^-1^ and increased with photoperiod, as more electricity was required to supply more hours of light and to maintain appropriate climate conditions, including removal of heat generated by the lamps. Among the components of electricity supply, lighting accounted for 41% to 63% and air climatization 23% to 31% (see Supplementary Materials 2.2).

The results for other LCIA categories related to ecotoxicity were similar to specific GWP, with lower values obtained for higher CO_2_ concentrations and photoperiods, as presented in the Supplementary Material, Section 2.3. The conditions that minimized specific GWP (TPF = 20 ℃, [CO_2_]PF = 3,300 ppm, PP = 24 h d^−1^) also led to the minimum specific values for Freshwater Ecotoxicity (FE), Marine Ecotoxicity (ME), Terrestrial Ecotoxicity (TE) and Human Toxicity (HT) which were 0.28, 0.38, 17.05 and 3.64 kg 1,4-DCB (kg FW)^−1^, respectively. In these categories, the marginal increase in yield was greater than the marginal increase in emissions associated with electricity and CO_2_ consumption, demonstrating that the environmental conditions that maximize resource consumption and yield can also minimize environmental impacts. The largest contributors to these impact categories are again the seed trays and consumables, infrastructure, and electricity consumption. Notably, the transport of CO_2_ supply became relevant for TE.

## Discussion

The field of LCA research into the application of ag-tech such as IVF systems in UA demonstrates a wide range of results, with reported emissions ranging from 0.01 to 54 kg CO_2_e/kg to produce different leafy green crops^15^. Disparities are often justified as a result of the type of crop analysed, the combination of ag-tech installed, and the location^6,12,18^. However, most studies use fixed operational conditions for conducting the LCA, which may correspond to either the actual conditions used in the specific IVF system analysed or the assumed desirable conditions for production^8,16^. In contrast, our study showed that varying a farms operating conditions alone, even while using a single IVF system to grow a single plant species, can result a specific GWP from 3.3 to 63.3 kg CO_2_e/kg^−1^. Our results show that, by managing the temperature, CO_2_ concentration, and photoperiod to achieve maximum capacity, the farm produces the highest yield and has the lowest impacts in specific GWP. These findings highlight a critical area of focus for UA research on ag-tech and IVF systems: understanding the range of effects of managing any IVF system to enable better decision-making for production goals and environmental performance. Dynamic and prospective analysis, as carried out in this study, can facilitate the development of IVF management plans that improve the environmental performance of the farm while increasing revenues.

The optimal conditions for minimum specific GWP coincided with those of maximized yield, with a maximum yield of 290.5 kg week^−1^ achieved at 20 ℃ for 24 h of continuous lighting and the highest possible CO_2_ concentration. However, these conditions also correspond to the most intensive resource consumption, with maximum photoperiod and CO_2_ concentration, regardless of temperature. By increasing resource consumption, the marginal increase in yield was higher than that of absolute GWP, indicating that intensive production is the best way to minimize GWP per unit weight of production. This trend held true for all other impact categories studied as well. Although this result may seem surprising in conventional farming, where environmental optima do not always coincide with maximum production intensity^2^, our study defined an option space of plausible operational conditions using IVF. At the point of maximum yield, returns of input use on yield are approximately zero but those inputs have an environmental cost, and therefore it may be preferable to produce less. An inversion point where returns of input use start to become negative were naturally outside the domain of those plausible for the variables studied. There would surely be a limit to the gains brought by intensification in cases where the research expanded the option space, but that limit lies beyond the option space assessed in study.

To assess if the main assumptions of the study had influenced the conclusions, we used two main strategies. First, we checked whether the plant model used provided us with results that are qualitatively well supported in the experimental literature for kale microgreens. Second, we assessed whether we could be underestimating the environmental impact of production and use of one critical input, namely industrial CO_2_ consumption.

Regarding the qualitative relationship between the model variables and kale microgreens yield, we found evidence that continuous lighting results in maximum yield, as past literature has found that increases in photoperiod lead to increases in kilograms grown^23,24^. Kale has been found to maximize its production for the largest photoperiod possible^21^, a characteristic shared with other crops, such as gai lan^25,26^, and sugar beet^27^, among many other species^28,29^. In addition, our plant model showed that increases in CO_2_ concentration always increased yield, which is consistent with the literature^30–32^. The temperature was also found to affect yield, with 20℃ resulting in the largest yield^21,30^. Similarly, our results suggest that the optimal conditions for maximum yield also involve intensive resource consumption, with the highest possible photoperiod and CO_2_ concentration, regardless of temperature. The limitation of the IVF system, as in all farming systems, is the availability of lighting for photosynthesis. Due to the fact IVF systems can apply 24 h per day of lighting, the technology offers a major advantage to the maximum yield while reducing environmental impacts of each unit produced through strategic planning and operational control.

While intensive production resulted in higher yields, the total environmental impact increased as well, highlighting the need for careful decision-making in the management of IVF operations. Our study provides a range of plausible operational conditions that can be used to optimize both yield and environmental performance, enabling the development of IVF management plans that improve the sustainability of indoor vertical farms. It is important to note that our plant model does not include effects due to changes in relative humidity nor yield decrease due to excessive increases in CO_2_ concentration, which could potentially become detrimental^33^. We assumed that CO_2_ increases, when combined with fertilization, can lead to biomass growth regardless of initial concentration^30^. None of the articles mentioned have increased fertilizer supply with increases in CO_2_ concentration, and as such the reduced effect of CO_2_ enrichment on plant growth might be due to lack of fertilizer in studies described.

The current work found through the plant model increases in CO_2_ concentration always increased yield. In the literature, the relationship between CO_2_ enrichment and photosynthetic rate was found to increase the yield up to a given point. It is known that for early life stages in plants growth is exponential, allocating resources such as CO_2_ into increasing biomass in an ever-faster pace^34,35^, before processes of maturing start to take over^36^. This result may be understandable given that the study focused on growing kale microgreens, using a short growth cycle of 1 week for germination and 1 week for growth inside the IVF which corresponds to early stage of growth of the plant^37,38^. However, it is also true that maximum photoperiod can damage some plant species^21,39^. Injuries manifest as yellowing, necrosis, or drying of leaves when plants are subjected to extended photoperiods. The use of increased photoperiods can lead to photosynthetic rate decrease and disturbances in the plants’ circadian rhythm, which jeopardize plant vitality, yield and quality^40^. These effects may vary depending on the plant species and age at harvest, and as such, future research should test the intensification of microgreen production applying a range of settings.

We investigated industrial CO_2_ supply further to identify whether we underestimated the role of CO_2_ supply in the plant model. Plants require CO_2_ as fertilizer when exposed to light and naturally store it during dark period which affects CO_2_ concentration in the air^6,16^. It had a low contribution to GWP results, in that the increases of GWP were small when CO_2_ concentration in the IVF increased. Therefore, it always paid off to provide more industrial CO_2_. To confirm the validity of our finding, we investigated several avenues. Initially, we examined the possibility that the specific GWP of CO_2_ supply may have been underestimated. The process used from the ecoinvent 3.8 database to represent liquid CO_2_ sourced from chemical manufacturing plants, and therefore did not carry any environmental burdens. The only environmental impacts associated with the CO_2_ were due to the processes required for its liquification and transportation, including the replacement of cylinder refills. Here we attributed a specific GWP of 1.5 kg of CO_2_e per/kg of industrial CO_2_ and in the literature the GWP of CO_2_ supply has been found to indeed be negligible^41^, with a specific GWP of 0.12 kg CO_2_e per kg CO_2_^–1^ at production gate^42^, and of 0.91 kg CO_2_e per kilogram of CO_2_ used in an IVF^43^. All these values are smaller than the one used here. As such, the hypothesis of the specific GWP of CO_2_ supply being underestimated could be discarded.

Leakages were then introduced in the IVF mass and energy balance, to account for events where even at low ventilation rates, most CO_2_ supplied to the IVF can be lost due to accidental ventilation, generally, when the IVF door is open CO_2_ is lost instead of being absorbed by the plants^19^. When recommend CO_2_ concentration for plant growth is set at 1,000 ppm, and higher concentrations leads to larger CO_2_ leakages within the exterior of the IVF^33,45^. Leakages caused an increase in absolute GWP, as this would not only require an increase in CO_2_ supply to compensate the CO_2_ escaping the farm, but also lead to direct emissions of CO_2_ to the atmosphere. All results presented here include leakages, and yet they still led to the optimum conditions in the farm being at maximum CO_2_ concentration^46^. The air changes per hour in the IVF had to be of 15 h^-1^ (870.8 m^3^ of air leaked per hour), three orders of magnitude higher than the value considered, for GWP to start increasing with industrial CO_2_ supply.

Electricity consumption had a large contribution to GWP (35% to 54%), and indeed GWP increased as photoperiod increased. These results were expected based on past research findings^12–14^. The contribution of seeded trays to GWP was found to be substantial, accounting for 30% to 46%, with seed production being the second largest contributor. This finding is consistent with previous studies^16^, but many research studies consider seed production as negligible^47^. By intensifying production, higher yield is obtained from seeded trays, diluting the relative contribution to GWP tied to the seeded tray used. Maximizing yield is critical for reducing the relative contribution of seeded trays to GWP, as more yield is obtained from the same seeds, tray, and substrate. It is essential to have a process base plant growth model that drives all LCI parameters dynamically to achieve the optimum yield and understand the environmental impact^48–50^. Therefore, the importance of seed production should not be neglected in future studies, and plant models should be incorporated to improve the accuracy of LCI parameter definitions.

This study breaks new ground by evaluating the optimal settings for managing IVFs to minimize emissions of food produced. In this sense, it is to our knowledge the first of its kind because, in contrast to previous research, this study assesses a comprehensive range of plant growth conditions that directly impact yield and energy use. What sets this study apart is the incorporation of process-based model for plant growth into the LCI of the IVF, allowing for a dynamic evaluation of the effects of plant growth conditions on demand for consumable inputs^31,33^. By exploring the full growth potential of the IVF, the plant growth model did not limit or fix the LCI data for consumable inputs required for producing the functional unit. The process-based model simulates IVF operations on a weekly basis, providing an accurate representation of reality, and LCI data consumed by each process is automatically incorporated into the results^45^. This approach allows for changes to key variables such as photoperiod, temperature, and CO_2_ fertilization and assessing the resulting effect on yield and environmental impact indicators. This evaluation of the optimal settings for IVF is critical for UA in the face of increasing food demand and climate change. By minimizing emissions and exploring the full growth potential of IVFs, this study offers a promising solution for sustainable food production that can meet future demand while minimizing environmental impact.

Finally, future research should assess the consequences of environmentally optimized production for the profitability of IVF farms. Here we showed that 290.5 kg week^−1^ of kale microgreens can be produced at 20 ℃, using the highest photoperiod and CO_2_ concentration. The lowest yield grown was 12.9 kg CO_2_e week^−1^ at 17 ℃ with minimum photoperiod and CO_2_ concentration. The difference was therefore 277.6 kg week^−1^ or, to put it another way, a reduction of 3 ºC, 91% in CO_2_ concentration, and 66% in photoperiod reduced 95% of the yield. Therefore, increases in resource consumption seem to increase yield more than proportionally. This suggests that the environmental optimum can also be expected to maximize gross revenue as measured by the difference between revenue from microgreen sales minus variable costs with resources used. For this IVF and for kale microgreen production, we expect from the results that maximum intensification could be a win–win for environmental and business sustainability. However, in this study we were unable to analyze economic and social aspects of IVF management as this was a study of prospective IVF that is not yet operational. Real costs were unknown, as well as revenue, which is even more difficult to estimate as it changes locally and is highly dependent on the business model—direct sales to customers or businesses. We therefore suggest this economic cost–benefit analysis and social impacts of management for future studies that focus on active urban IVF businesses.

## Conclusion

The present study investigated the impact of plant growth conditions on the environmental performance of a building-integrated IVF system in an UA context. Our study is the first to use a process-based model to analyse an option space of operational parameters for IVF management in an LCA study. We used a dynamic LCI model to simulate the consumable materials response to changes in plant growth conditions, such as CO_2_ concentration, temperature, and photoperiod. Our results indicate that the specific GWP of the system varied significantly depending on operational conditions and that IVF management can contribute to a wide range of GWP results, even when producing the same crop with the same combination of ag-tech. These findings offer an explanation for the wide variation of LCA results reported for CO_2_e/kg of leafy greens in past studies of UA. We also found that intensification of production was environmentally beneficial in the IVF system, as conditions of minimum specific GWP and other impact indicators coincided with maximum use of resources and yield.

We observed that the marginal increase in yield due to increased resource use was larger than the marginal increase in all impacts. Our study focused on microgreens, which are in an early stage of growth and show a good response to resource abundance. Operating the IVF system at full capacity not only improves environmental performance but may also potentially provide economic benefits for IVF businesses and managers. Therefore the findings of this research address the gap in literature, by informing IVF researchers and managers about the range of optimum conditions for improving environmental performance for reduce the effects of urban food production on climate change.

Future research should adopt similar modelling frameworks for other IVF systems, plants, and locations, as changes in the plant development cycle and ag-tech used could produce different results from the ones obtained here. These studies should also include an economic cost–benefit analysis and social impact evaluation to assess if environmental optima are positive or detrimental to business sustainability. In summary, our study provides insights into the potential for IVFs to improve their environmental performance through optimization of plant growth conditions and highlights the need for further research in this area to promote sustainable food production in the face of climate change.

## Methods and materials

### Case study description

In this study, we utilized LCA, a framework that assesses potential effects on the environment and resources utilized in a production system^51^. Specifically, we applied LCA to evaluate an IVF installed in the Lisbon region but not yet being commercially exploited. We, therefore, carried out a prospective evaluation, i.e., an LCA used for novel systems or technologies where data are scarce. This study is in accordance with the LCA requirements of ISO Standard 14,044; 2006 where the LCI stage uses mass and energy process-based modelling to build unit processes for plant growth and climatization within the unit and the database ecoinvent 3.8 to determine emissions for background processes^48^. We used the software OpenLCA version 1.11.0 to calculate unitary emissions for each equipment and material input^8,52^. Emissions were imported into MATLAB version R2019a^53^, where the process-based and mass-balance modelling was carried out and final results compiled.

**Figure 4:**
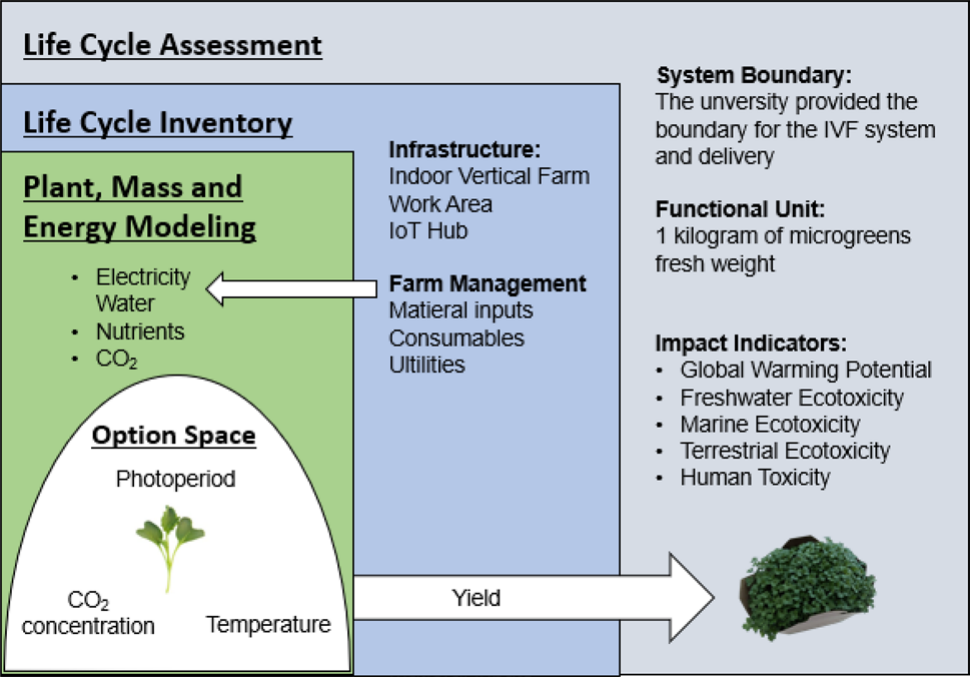
Schematic depiction of the analysis framework; Life cycle assessment where the life cycle inventory was based on plant, mass, and energy process-based modelling with a variable set of parameters explored as an option space.

Figure 4 illustrates the procedure for the analysis that will be detailed in the following sections. We used an LCA framework (represented in grey). The LCI (in blue) involved the development of a plant model that estimates yield under a set of operational conditions that define an option space for IVF management, as well as the mass and energy balance of the IVF (in green).

The IVF modelled here was a prospective building-integrated technology that included a 32 sqm growth chamber with vertical hydroponics and LED lighting system installed inside the technical area of a building on a university campus located in Carcavelos, Lisbon Portugal. Detailed specifications for the infrastructure and combination of ag-tech used in the IVF system (growth chamber, LEDs, and growing systems) were taken from an article by Parkes et al.^16^. We combined that LCI for the infrastructure (namely the IVF and preparation work area) with adaptations made to estimate the impacts of electricity consumption and additions made to climatization (heating, cooling, and humidity control) for the modelling of dynamic plant growth, as outlined in 5.2.

### Goal and scope

The goal of this study aimed to investigate the impact of various operational parameters on microgreen growth and environmental performance within an IVF. The study explored an option space for the management of IVF growing microgreens, where the functional unit was 1 kg of fresh weight kale microgreens supplied locally to the campus. The weekly production was modelled as the average over a 12-month period with a single growth cycle requiring 2 full weeks from seeding to harvest. Life cycle inventory materials were introduced to the process-based plant model as a 1-week cycle of growth, after 7 days of germination. Known as a leafy-green, kale (*Brassica oleracea, var. acephala*) has published data on the early growth stage, and when grown for 7 to 14 days after germination, the harvested sprouts, and first leaf’s final product is a microgreen with high nutritional value and cultivation density^35,38^.

Methodologically, we aimed to address the static nature of LCAs regarding the production system’s response to varying conditions inside the IVF, as previous studies consider yield and material inputs as fixed in the LCI^54,55^. To do so, we use process-based modelling to determine the dynamic relationship between material inputs, environmental conditions, and the response of plant growth inside the IVF. To reduce some complexity, the variables explored relate to those conditions with high impact on energy consumption and under the direct control of IVF management, specifically operating hours of LEDs known as photoperiod, CO_2_ concentration (associated with fertilization), and air temperature^14,57^. To investigate the relationship between maintaining ideal growth conditions for crops, yield, and environmental performance, a dynamic LCI was produced in this study. This approach allowed for an exploration of the material input requirements for crop demand and the impact on the final functional unit.

### Life cycle inventory

This section describes the fixed variables and the database processes used to estimate their environmental performance (2.2.1), how this LCI was combined with the process-based plant model (2.2.2), and the mass and energy balances of the IVF and subsequent consequences on LCI material inputs (2.2.3). Unlike previous studies, the LCI considered in this study needed variable components for inputs tied to climatization and plant growth for inclusion in the process-based model to explore the option space available for plant growth. Depending on the photoperiod, air temperature, and CO_2_ concentration inside the IVF, the process-based model estimated the crop yield, water and fertilizer supplied, and the CO_2_ and heat flow exchanges between the plants and their environment. Based on this internal system dynamics, and through mass and energy balances, the required moisture, CO_2,_ and electricity to maintain air humidity, CO_2_ concentration, and temperature were calculated. The yield and the required inputs for plant growth and climatization for the conditions assessed could then be included in the inventory.

#### Inventory for the IVF

Data for IVF infrastructure, equipment and energy, and material inputs for production were sourced primarily from the ecoinvent v3.8 and Agribalyse v3.0.1 datasets^48–50^. The infrastructure of an IVF generally combines three ag-tech: a controlled environment growth chamber, a vertical soil-less growing system, and a lighting system^12,16^. This LCI includes a LED system for lighting, a vertical hydroponic growing system, and the climate-controlled growth chamber. Those systems require installation materials, LED fixtures, steel structures, trays, climate control equipment, transportation of materials/equipment, and IVF assembly. Key soil-less growing equipment included piping, filters, tanks, nutrient dosing, and water pumps. Additionally, an electrical hub was introduced for LED lighting, climate control, and sensor integration with the associated cables and electronic components. Due to the data requirement of the building-integration in the selected case study an IoT main hub and sensor clusters were all included in the infrastructure. The work area used for operating IVF processes for seeding, harvest, and packaging required a work room with similar inputs as the climate growth chamber. The inventory includes an updated energy mix to improve the accuracy of electricity supply in Portugal, the equipment required for CO_2_ fertilization, the liquid CO_2_ dosing via aluminium cylinders, and the humidifiers for relative humidity control (see Supplementary Material, Sections 1.5.1 to 1.5.5). The majority of IVF infrastructure used to produce the functional unit has a 20-year lifetime applied, whereas 10-year lifetime was considered for the LED lights and the growing trays^58^. All LCI inputs for a cradle-to-gate system boundary were defined, similar to the circular supply proposed for the university campus scenario by Parkes et al.^16^. Table 1 summarizes the data sources of equipment and materials in the LCI required to execute the growing process for weekly production of kale microgreens for harvest and sale and treatment of the by-product output of compostable biomass.

**Table 1:** Life cycle inventory for the weekly production of fresh weight of kale microgreens.

Name	Unit	Source
Inputs—fixed		
Indoor Vertical Farm	Items	Econinvent 3.8^16^
Cleaning	Items	Econinvent 3.8^16^
LED Lighting	Items	Econinvent 3.8^16^
Seeded trays	Items	Econinvent 3.8, Agribalyse 3.0.1
Inputs—dynamic		
Supplied CO_2_	kg	$Plant\, model, \,econinvent \,3.8, \,mass\, and\, energy \,balance$
Water	kg	$Plant \,model, \,econinvent \,3.8,\, mass\, and\, energy\, balance$
Electricity	kWh	$Econinvent \,3.8,\, mass \,and \,energy \,balance59$
Ready for harvest kale	kg	Plant model
Outputs		
Excess CO_2_	kg	Plant model, econinvent 3.8
Kale Microgreens	kg	Plant model, econinvent 3.8
Compostable biomass	kg	Plant model, econinvent 3.8

Life cycle inventory data sources applied in the study are grouped by system inputs as either fixed or dynamic and outputs including infrastructure data from Parkes et al.^16^, datasets from ecoinvent 3.8 and those produced by the process-based models.

Processes for IVF operations were replicated in OpenLCA to determine the material inputs consumed to produce the functional unit and the impact of these materials on the environmental performance of the technology. The system boundary incorporates 4 processes described below: Seeding, Growing, Harvesting, and Cleaning. All IVF operations for this study are based on a weekly production cycle. It begins with the seeding process, which produces the seeded tray for germination and growing. The material inputs for the process are based on every kilogram of fresh weight produced and include: 0.07 kg of seeds equivalent to conventional cauliflower seeds in Agribalyse database, 3 kg of coconut fibre with husks for the substrate, plastic sheets, and polyethylene reusable trays. Due to requirements for food safety and sanitization, the IVF requires constant cleaning. We thus included cleaning at the end of the seeding, harvesting, and packaging processes. Cleaning was estimated to take a maximum of 2 h for each functional unit, including soap and water, plus clothes, gloves, and glasses with a 1-year lifetime.

Transferred from the preparation work area, the seeded tray then enters the growing process inside the IVF, where it enters the dark layer without LED light, which is the germination tier of the vertical hydroponic system. Following 7 days, sprouted seeds appear and the trays are moved upwards into the cultivation tier where LED light, water, and nutrient dosing are controlled by interval set points. A flowrate controller was also considered to manage the supply of CO_2_ to the inside air. For the growing process nutrients are defined as an NPK mix of 15-15-15 supplied by dosing 1 L of water with 15 mL of nutrient mix and the closed loop system produces no waste. The data for consumables supply and delivery are presented in the Supplementary Material, Section 1.5.6.

Operating the IVF requires electricity as a major input for all systems involved in the growing process, from circulating water and nutrients to the energy demand specific for running the climate system and managing indoor environmental conditions. Indoor conditions are changed by controlling temperature (heating and cooling), alternating hours of light from LED (photoperiod), and changing internal atmosphere conditions via humidifiers, air ventilation, and fans. All seeded trays spend a total of 7 days in the cultivation tiers where these conditions were used by the process-based model to predict the effects of changes in these variables on electricity consumption, yield produced and impact indicators.

The energy mix considered in ecoinvent is representative of Portugal in 2018 and was therefore updated with the average production mix available in Portugal between January and August of 2022: 34.1% natural gas, 6.4% fossil CHP, 11.1% hydro, 33.4% wind (with storage), 8.7% bioenergy and 6.6% solar^59^. Electricity transmission network inputs and emission of sulphur hexafluoride for the voltage conversion process were included. The process considering the different forms of electricity production is presented in Supplementary Material, Section 1.5.1.

After completing 14 days of growing process in the IVF growth chamber, each seeded tray is ready for harvest^37^. Microgreen shoots and leaves are separated from the roots and substrate in each tray, where the fresh cut kale microgreens are packaged into reusable boxes for delivery on the campus. Leaving the roots and substrate as organic waste, a co-product destined for treatment to create compost for direct use on university gardens with zero emissions, as new compost purchased for campus is avoided. The quantities considered are calculated based on the total fresh weight of microgreens and roots (kg), plus the mass of the substrate as 3 kg per kilogram of kale microgreens.

#### Process-based model for plant growth

**Figure 5:**
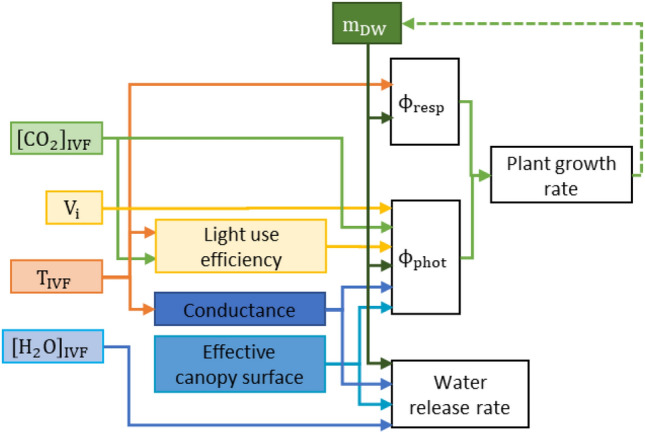
Simplified schematic depiction of the process-based plant model. All variables; CO_2_ concentration ([CO_2_]_IVF_), air temperature, (T_IVF_), light intensity, (V_i_), and humidity ([H_2_O]_IVF_), influence directly or indirectly via light use efficiency and conductance, photosynthesis (ϕ_phot_), respiration (ϕ_resp_) and growth rate of plant dry mass, (m_DW_).

We used the plant growth model by Van Henten^30^ to estimate the growth of kale microgreens produced inside the prospective IVF technology studied. The model was implemented in MATLAB R2019b^53^. The original model simulates the growth rate of lettuce (*Lactuca sativa L.*) with some parameters designed for this species' full growth cycle, while other parameters were either physical constants or depended on the environmental conditions available for the plant. Though designed as a continuous model, it is applied with discrete intervals based on the farm design^31^. Figure 5 illustrates the relationship between these IVF conditions and the respective plant growth processes of photorespiration and photosynthesis. The growth rate is a function of the efficiency of conversion from CO_2_ to mass, and the difference between the CO_2_ intake during photosynthesis and CO_2_ released during respiration.

The time step considered in the plant model was 15 min to match IVF management frequency for setting fixed conditions in the farm. Production assumed 7 days of germination, followed by 7 days of growth under lights and then harvesting. Based on this growth cycle the model was changed to use the total photon flux density and photosynthetically active radiation efficiency for lighting (see Supplementary Material, Section 1.2). The original model did not consider water and fertilizer demand as it assumed plants grew in unlimiting conditions. Updates were made to use a unitary transmission coefficient, a dry mass content ratio, calculation of moisture and heat exchange between plants and air; and fertilizer, industrial CO_2,_ and water consumption of plants based on nutrient content. As production is continuous in time, the whole IVF system capacity is 126 trays used in 1 plant growth cycle, but not simultaneously. One-seventh of the trays begin growth every day, ensuring that every day 18 trays are available for harvest, producing a total of 126 trays harvested per week. The individual mass and energy flows were then also translated in time and summed to result in total, continuous flows.

The lettuce-specific parameters were then updated to be representative of kale microgreens. Calibration for kale occurred as a result of selecting a species for production as microgreens and based on the availability of published data for different plant growth conditions on this species. The calibrated parameters were those that played the largest effect in the model^30^, as presented in the Supplementary Material Section 1.1. Data from two papers were used, namely Ford and Thorne^31^ who measured kale growth evolution as a function of CO_2_ concentration and light intensity, and Chowdhury et al.^33^ who measured growth depending on temperature. As the papers used in calibration did not report growth per unit area, as used in the model, two extra parameters for calibration were considered, which intend to be the best estimates for the area of growth^31,33^. The calibration took place in MATLAB R2019b^53^ using the Optimization Toolbox (see Supplementary Material, Section 1.1).

The option space for growth was then determined and used to evaluate the effects on yield of various changes to IVF growth conditions. The option space studied targeted temperature of between 15 to 25 °C in steps of 1 °C, CO_2_ concentration of 400 to 3300 ppm in steps of 100 ppm, and photoperiod of 8 to 24 h d^−1^ in steps of 1 h d^−1^, respectively, resulting in 5610 possible combinations. These values were chosen from the available literature on kale production (Table 2).

**Table 2:** IVF conditions used for kale production.

$\mathrm{Temperature}$ ($\mathrm{^\circ{\rm C} }$)	CO_2_ ($\mathrm{ppm}$)	$\mathrm{Photoperiod}$($\mathrm{h }{\mathrm{d}}^{-1}$)	Reference
15 to 20	300 to 3300	16	^31^
14 to 26	400 to 1600	16	^33^
15 to 21	Uncontrolled	12 to 24	^60^
16 to 24	50 to 1200	16	^32^
20	Uncontrolled	6 to 24	^21^
17 to 21	470	16	^39^

This table are the variables used to define the options space conditions of the indoor vertical farm for temperature (°), CO_2_ concentration (ppm) and the photoperiod hours as per day (h d^−1^).

#### Mass and energy balance of the IVF

We calculated mass and energy transfers between the IVF and the building, to determine the heat, humidity, and CO_2_ exchange of the farm with the outside climate in the technical area. We considered climatization leakage, i.e. loss of CO_2_, heat, and humidity to the exterior of the IVF due to day-to-day operations^19^. This includes heat exchanged through the growth chamber walls, released by human workers or functioning equipment, and absorbed by the plants^6,61^.

Leakage frequently creates an imbalance in calculations due to loss of atmospheric conditions through opening doors or changes in IVF conditions^45^. The heating, cooling, humidity, and CO_2_ are added or removed via control of climate systems, which were all calculated by the mass and energy balances of the IVF system. Based on the maximum air flowrate of the climate system, the desired air flowrate and CO_2_ concentration were calculated. The water mass balance was then calculated to consider water released by human workers and by the plants, which enters the air supply as moisture that was added and/or removed^46^. Once all mass exchanges were calculated, a heat balance was determined. In this study, the calculations developed in the model consider three main imbalances (found for CO_2_, moisture, and heat flows due to leakage) that were corrected. Calculations can be found in the Supplementary Material in Section 1.4.

### Life cycle impact assessment

For LCIA we explored five main categories of environmental impact: Global Warming Potential (GWP), Freshwater Ecotoxicity (FE), Marine Ecotoxicity (ME), Terrestrial Ecotoxicity (TE), and Human Toxicity (HT)^20,22^. Calculations of the environmental impacts of each component of the farm were executed in OpenLCA, version 1.11.0^8,52^ making use of the ReCiPe 2016 (H) impact assessment method. Results were then imported to MATLAB, version R2019a^53^. Knowing the impacts of fixed inputs, such as the infrastructure and seeded trays, and dynamic inputs, such as electricity, water, and nutrient consumption, the overall impacts were calculated as the sum of the impacts of each input for all the option space in a much shorter time, than if all calculations had been performed in OpenLCA.

## Supplementary Information

Supplementary Information.

## Data Availability

The data that support the findings of this study are available from Canguru Foods LDA but restrictions apply to the availability of these data, which were used under license for the current study, and so are not publicly available. Data are however available from the authors upon reasonable request and with permission of Canguru Foods LDA.
