# Supplementary material for: Life cycle assessment of microgreen production: effects of indoor vertical farm management on yield and environmental performance
Source: Sci Rep. 2023 Jul 13;13:11324. doi: 10.1038/s41598-023-38325-0 (PMC10345114; doi:10.1038/s41598-023-38325-0)
Supplement: Supplementary file 1 — Supplementary Information. [file 41598_2023_38325_MOESM1_ESM.docx]

Supplementary material: Manuscript SREP-23-00322,

‘Life Cycle Assessment of microgreen production: Effects of Indoor Vertical Farm management on yield and environmental performance’

S.1. Methods

We aimed to identify a plant model with the capacity for predicting the growth of a microgreen and produce results for an equivalent species to that of Parkes et al.^3^. In the absence of plant yield measurement and the dynamics of how it is quantitatively affected by operational conditions, a model for plant growth was used. The model chosen was developed by Van Henten^4^. It has the growth rate depending on the plant mass dry weight ($m_{DW}$), and the plant farm’s air temperature ($T_{IVF}$), humidity ($\left[ H_{2}O \right]_{IVF}$), CO_2_ concentration ($\left[ CO_{2} \right]_{IVF}$), and light intensity ($V_{i}$).

Van Henten’s model simulates the growth rate of lettuce (*Lactuca sativa L*.) and, as such, some of the parameters are designed for this species, while others are either physical constants or depend on the environmental conditions available for the plant. Dry weight (DW) growth rate is a function of efficiency of conversion from CO_2_ to dry mass, and the difference between the CO_2_ intake during photosynthesis $\phi_{phot}$, and CO_2_ released during respiration $\phi_{resp}$. The calculation of these quantities was done as presented in the original work, where Van Henten designed the model continuously and was applied discretely based on the greenhouse design, depending on the desired variables being studied^4^. Time discretization was done through first order backwards differencing to calculate plant growth. In mass and energy balances, time derivatives were assumed as null to allow the calculation of the required mass and heat supplies/removals to ensure mass and energy equilibrium.

The calibration of the plant model for kale happened with resort to data published in the literature for different plant growth conditions, as described in section S.1.1.

Changes to the plant model, which can be found in section S.1.2., were required to take into account the design of the indoor vertical farm (IVF) considered, and the data found in literature for the calibration.

Microgreen production was assumed as comprising 7 days of germination followed by 7 days of growth under lights and then harvesting. As the model required the knowledge of an initial dry mass point, the value of $6\times{10}^{-3}\mathrm{kg}m^{-2}$ was assumed based on the growth test in the microgreen product development stage. As the model did not consider water and fertilizer demand, it was assumed plants grew in unlimiting conditions of water and fertilizer supply. The production cycle considered was of one week, at the end of which all 126 trays have been harvested. It is assumed a seventh of the total trays is harvested every day, and as such, the seven groups of 18 trays begin growth one day after the previous group.

The calibrated plant model was used to evaluate the options space and feasibility of various changes to IVF growth conditions and subsequent effect on LCI inputs. The option space studied targeted temperature of 15 to 25 ℃ in steps of 1 ℃, CO_2_ concentration of 400 to 3300 ppm in steps of 100 ppm; and photoperiod of 8 to 24 h d^-1^ in steps of 1 h d^-1^, respectively, resulting in 5610 possible combinations. These values were chosen from the available literature on kale production (Table 2 in the main text).

S.1.1. Calibration procedure

The plant model used in this study was based on that of Van Henten^4^. Given the original model was calibrated for the growth of lettuce, data on the growth of kale from the literature had to be used to calibrate the model for the growth of microgreens. According to Van Henten’s verification work^5^, the parameters which play the largest effect in the model, and therefore were considered in the calibration, are the yield factor $c_{\beta}$, the light use efficiency at high CO_2_ concentrations $c_{\varepsilon}$, the coefficients relating temperature to carboxylation conductance of CO_2_ through the leaves $c_{car,1}$ and $c_{car,2}$, the light extinction coefficient $c_{k}$, and the leaf area ratio $c_{lar}$. The third coefficient relating temperature to carboxylation conductance of CO_2_ through the leaves $c_{car,3}$ was also considered in the calibration as it also affected how CO_2_ conductance responded to temperature changes. The rest of the parameters of the model kept their original values, aside for those mentioned in section S.1.2.

Calibration made use of data of two studies on kale growth, as this was the plant species with the most available data: Ford and Thorne^1^, and Chowdhury et al.^2^.

The data used from the work of Ford and Thorne ^1^ were those presented in table 1 and 2 of the article. Light intensity was converted from $\mathrm{cal} dm^{-2}\mathrm{mi}n^{-1}$ to $W m^{-2}$. Given these are in the original units used by the plant model, it was used directly with no conversion to photosynthetically active radiation (PAR). The initial dry mass of experiment 2, whose results are presented in table 2 of the article, is unknown. This experiment happened 25 days after sowing, and in the 25 days leading to the experiment, kale grew under the same conditions of the initial stage of experiment 1. In experiment 1, the initial weight was of 0.4 g DW, measured 45 days after sowing. As an estimation for the initial DW for experiment 2, it was assumed the growth rate was constant and equal to that of experiment 1, with a value of 0.4 g DW/45 days = 0.0089 g DW day^-1^, resulting in an initial DW for experiment 2 of 0.22 g DW after 25 days.

From the second article ^2^, only data from experiment 1 was used, whose results are presented in the article’s table 4, as the plant model used does not include effects on plant growth due to changes in relative humidity nor yield decrease due to increases in CO_2_ concentration. As in this article the reported weight is in a fresh mass basis, DW content was estimated as a function of time, as shown in Equation $(2)$ below. Also lacking the initial weight, it was assumed the plants had a fresh weight of $2 g$ three weeks after sowing, a value based on the appearance of the plants from the article’s figure 3. Conversion from photosynthetic photon density flux to power flux was made using a conversion value of $2 \mu mol J^{-1}$ ^6^.

As the values for yield in both works are not in mass per unit area, but in plain mass, two values for area were considered to be representative of the area of the pots used in either work: $A_{1}$ for Ford and Thorne’s work^1^, and $A_{2}$ for Chowdhury et al.’s work ^2^. However, these values are unknown. As such, they will be included in the calibration process.

The error function to be minimized during calibration is the following,

|  | $f_{er}=\sum_{i=1}^{22} \left( w_{i}\sqrt{\sum_{j=1}^{n} \left( \frac{x_{i}-\hat{x}_{i}}{x_{i}} \right)^{2}} \right)$ | $( SEQ Eq. \backslash* ARABIC 1)$ |
| --- | --- | --- |

where $f_{er}$ is the error function ($\mathrm{kg}m^{-2}$), dependent on the squared differences between the DW per unit area reported by the articles $x$, and the value estimated by the plant model $\hat{x}$, for the 22 data points $i$. The parameter $w_{i}$ is a weight factor to prioritize the effects studied in each group of data. As the effect of temperature studied in the second article was considered worth conserving, the weight factor had a value of $20$ for those data points, being unitary for the remainder. The value of 20 was arrived to through trial-and-error.

The calibration routine was then called using the MATLAB function “fmincon”, that tests different sets of values for the parameters in order to minimize the error function $f_{er}$, starting from the initial guess of the set of values first supplied. The initial values chosen for $c_{\beta}$, $c_{\varepsilon}$, $c_{car,1}$, $c_{car,2}$, $c_{k}$, $c_{lar}$ and $c_{car,3}$ were the ones proposed by Van Henten ^4^, presented in table 3.1 of the work. The initial values assumed for areas of pots used in the experiments of Ford and Thorne ^1^, $A_{1}$, and Chowdhury et al. ^2^, $A_{2}$, were of $0.04 m^{2}$, $20\times20 cm$.

For the values of the parameters to retain physical meaning, upper and lower bounds were imposed for the use of the “fmincon” function. The bounds considered limited the values of the parameters within an order of magnitude above and below the values suggested by Van Henten ^4^. Exceptions to this rule were the bounds of $c_{\beta}$, which is bounded between $0$ and $1$, due to being a measure of efficiency; and $c_{k}$, which is bounded between $0.3$ and $0.9$, respectively for erectophile and planophile canopies ^7^ as is the case for kale. The areas $A_{1}$ and $A_{2}$ were constrained between $0.004$and $0.4 m^{2}$.

S.1.2. Changes to the plant model

$\boldsymbol{c}_{\boldsymbol{par}}$ **expressed as** $\boldsymbol{c}_{\boldsymbol{par}\boldsymbol{,}\boldsymbol{J}}$

One article considered to calibrate the model^2^ reports light intensity in units of $\mu mol m^{-2} s^{-1}$ and not in ${W m}^{-2}$, and the use of $c_{par}$, present in the original form of the plant model, requires the knowledge of the light spectrum of the light sources considered. As such, for the calibration process, the plant model was adapted to use $V_{i,PFD}$ as total photon flux density (PFD, $\mu mol m^{-2}s^{-1}$) and $c_{par,J}$ as the PAR efficiency of a given light source ($\mu mol J^{-1}$), instead of $V_{i}$ as total light power per unit area (${W m}^{-2}$) and $c_{par}$ as fraction of PAR of that power (dimensionless).

**No transmission coefficient since no radiation losses**

In the original plant model, a transmission coefficient $c_{rad,rf}$ was introduced to consider radiation losses from the sunlight through the greenhouse glass roof. In the current case, light falls directly from the LEDs on the plants, so $c_{rad,rf}$ is assumed as unity.

**Conversion factor from a dry to fresh weight basis**

As the functional unit used for the LCA is in a fresh weight (FW) basis, and not DW, a conversion factor is needed. A function for the DW percentage was built from published data on the growth of microgreen and baby leaves kale ^8^, and is presented below in Equation $(2)$, according to which the dry content increases as kale matures.

|  | $c_{DW}=\min\left[ 20\%,\max\left( 5\%, 0.3612\% t+1.983\% \right) \right], R^{2}=0.96$ | $( SEQ Eq. \backslash* ARABIC 2)$ |
| --- | --- | --- |

where $c_{DW}$ is the percentage of dry mass in the total weight at the time of harvest ($\%$); and $t$ is the age of the kale when harvested ($d$), that is, days between sowing and harvest. The minimum percentage considered is of $5\%$, whereas the maximum is of $20\%$.

**Moisture released and consumed by the plant**

The moisture release rate $\dot{m}_{w,plt,t}^{''}$ ($\mathrm{kg}m^{-2} s^{-1}$) is calculated according to the term presented in the water mass balance in the greenhouse climate optimization equations in Van Henten’s work ^4^, presented in Equation $(3)$ below.

|  | $\dot{m}_{w,plt,t}^{''}=\left[ 1-\exp\left( -c_{pl,d} m_{DW}^{''} \right) \right] \sigma_{H_{2}O} \left( \left[ H_{2}O \right]_{sat}-\left[ H_{2}O \right]_{IVF} \right)$ | $( SEQ Eq. \backslash* ARABIC 3)$ |
| --- | --- | --- |

where $c_{pl,d}$ is the effective canopy surface ($m^{2} kg^{-1}$), $m_{DW}^{''}$ is the DW per unit area at a given time ($\mathrm{kg}m^{-2}$), $\sigma_{H_{2}O}$ is the water conductance through the leaves ($m s^{-1}$), and $\left[ H_{2}O \right]_{sat}$ and $\left[ H_{2}O \right]_{IVF}$ are the saturation water concentration and water concentration in the farm air, respectively ($\mathrm{kg}m^{-3}$).

The variables $c_{pl,d}$ and $m_{DW}^{''}$ are calculated as shown in the work of Van Henten ^4^. Water conductance through leaves $\sigma_{H_{2}O}$ is calculated as presented below,

|  | $c_{stm}^{H_{2}O}=1.6 c_{stm}^{CO_{2}}$ | $( SEQ Eq. \backslash* ARABIC 4)$ |
| --- | --- | --- |
|  | $c_{bnd}^{H_{2}O}=1.37 c_{bnd}^{CO_{2}}$ | $( SEQ Eq. \backslash* ARABIC 5)$ |
|  | $\sigma_{H_{2}O}=\left( \frac{1}{c_{stm}^{H_{2}O}}+\frac{1}{c_{bnd}^{H_{2}O}} \right)^{-1}$ | $( SEQ Eq. \backslash* ARABIC 6)$ |

where $c_{stm}^{CO_{2}}$ and $c_{bnd}^{CO_{2}}$ are the stomatal and boundary layer CO_2_ conductances through the leaves, respectively ($m s^{-1}$), as presented by Van Henten ^4^.

The water mass rate consumed by the plants is calculated as the amount of water retained in the plant plus the amount of water transpired. This quantity is necessary for the life cycle inventory, and is calculated as presented below,

|  | $\dot{m}_{w,plt,c}^{''}=\frac{d m_{DW}^{''}}{dt} \left( \frac{100\%}{c_{DW}}-1 \right)+\dot{m}_{w,plt,t}^{''}$ | $( SEQ Eq. \backslash* ARABIC 7)$ |
| --- | --- | --- |

where $\dot{m}_{w,plt,c}^{''}$ is the water mass rate consumed by the plants per unit area ($\mathrm{kg}m^{-2} s^{-1}$), $d m_{DW}^{''}/dt$ is the DW growth rate per unit area ($\mathrm{kg}m^{-2} s^{-1}$), and $c_{DW}$ is the DW content ($\%$).

**Mass rate of CO_2_ consumed by the plants per unit area**

The mass rate of CO_2_ consumed and released by the plants for CO_2_ mass balance is calculated as

|  | $\dot{m}_{CO_{2},plt}^{''}=\phi_{phot}^{''}-\phi_{resp.}^{''}$ | $( SEQ Eq. \backslash* ARABIC 8)$ |
| --- | --- | --- |

where all units are per unit area, and $\dot{m}_{CO_{2},plt}^{''}$ is the mass rate of CO_2_ traded between the plants and the IVF air ($\mathrm{kg}m^{-2}$), $\phi_{phot}^{''}$ is the mass rate of CO_2_ absorbed by the plants during photosynthesis ($\mathrm{kg}m^{-2}$), and $\phi_{resp}^{''}$ is the mass of CO_2_ released by the plants during respiration ($\mathrm{kg}m^{-2}$).

**Nutrient concentrations**

The fertilizer necessary to supply the plants was calculated based on the nutrient content of the plants, estimated from published data and dependent on the age of the kale $t$ (d)^9^. The content of nitrogen is assumed constant and equal to $c_{n,N}=46.8 g \mathrm{kg}^{-1}$ of DW as an average of the values presented for different genotypes of kale at age $14$ to $16$ days after planting (assuming $7$ days of germination, results in $7$ to $9$ days after germination) ^10^.

|  | $c_{n,P}=min[10.7,\max(1.7,-0.1953 t+10.118)]$ | $( SEQ Eq. \backslash* ARABIC 9)$ |
| --- | --- | --- |
|  | $c_{n,K}=min[49.6,\max(8.3,-0.8442 t+51.338)]$ | $( SEQ Eq. \backslash* ARABIC 10)$ |
|  | $c_{n,Ca}=min[22.7,\max(9.5,-0.1258 t+18.722)]$ | $( SEQ Eq. \backslash* ARABIC 11)$ |

where $c_{n,P}$, $c_{n,K}$ and $c_{n,Ca}$ are the nutrient content per dry weight of phosphorous, potassium and calcium ($g kg DW^{-1}$).

S.1.3. Air conditions measured in the basement

The air temperature, CO_2_ concentration and relative humidity measured in the basement air with sensors are presented below in Supplementary Figure S1. The values were measured every 15 minutes, hence being the time resolution of the following mass and energy balances.

| 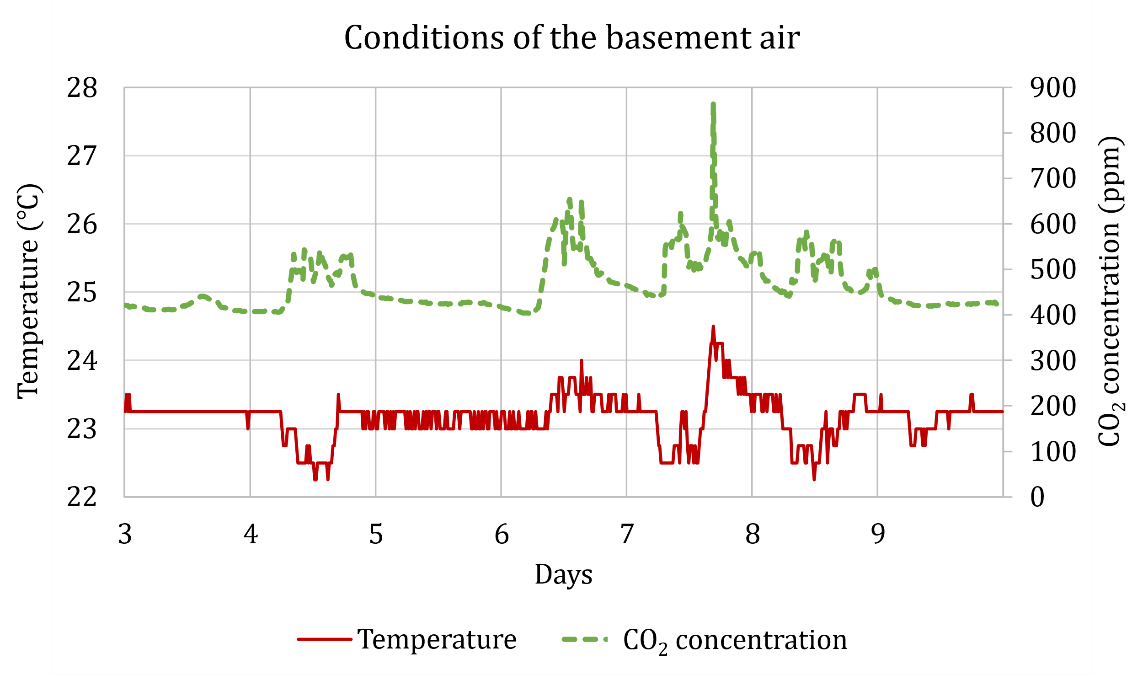 |
| --- |
| Supplementary Figure S1: Conditions of the air exhausted from the rooms from October 3^rd^, 2021, to October 9^th^, 2021. |

S.1.4. Mass and energy balances

S.1.4.1. Air leakage and air mass balance

Below are presented the equations used for leakage calculation. The air leakage out of the plant farm, $\dot{m}_{l}$, was calculated as the sum of two leakages: the leakage through cracks of $0.015 h^{-1}$ ^11^, equivalent to $\dot{m}_{l,ACH}=2.9\times{10}^{-4}\mathrm{kg}s^{-1}$, and the leakage through the door when it was opened by a worker $\dot{m}_{l,d}$. This door leakage was calculated as presented in Equation (12) as a function of the pressure difference between the plant farm and the basement air ^12^, which was considered to be $\Delta p=8 Pa$ ($0.0322 in_{H_{2}O}$)^13^.

|  | $\dot{m}_{l,d}=C_{A} A_{d} \sqrt{\Delta p} \rho_{air}\times\frac{1 m^{3}}{35.315 ft^{3}}\times\frac{1 min}{60 s},$ | (12) |
| --- | --- | --- |

where $C_{A}$ is an airflow coefficient ($cfm ft^{-2} in_{H_{2}O}^{-0.5}$), dependent on the number of people crossing the door without a vestibule per hour (and thus $\dot{m}_{l,d}$ was assumed to be the average flowrate in that hour); $A_{d}=17.97 ft^{2}$ is the area of the door; and $\rho_{air}$ is the air density ($\mathrm{kg}m^{-3}$). The two fractions on the right convert the units of $\dot{m}_{l,d}$ to $\mathrm{kg}s^{-1}$. The coefficient $C_{A}$ was approximated by a parabola defined by Equation (13).

|  | $C_{A}=-0.0024 N^{2}+3.7817,$ | (13) |
| --- | --- | --- |

where $N$ is the number of people crossing the door per hour ($h^{-1}$). It was considered $N=2$ in the time period defined by the 15-minute-long timestamps starting at 07:00 to 07:45, reflecting the entrance, presence and leaving of the human worker in the IVF between 07:00 and 08:00. That is the same period considered for the presence of the human worker in the IVF.

S.1.4.2. CO_2_ mass balance

As the supply fan has a maximum supply rate, the air supply rate $\dot{m}_{f}$ and leakage rate $\dot{m}_{l}$ are equal and maximized. Knowing the leakage rate out of the plant farm, the CO_2_ balance could be calculated.

|  | $V_{IVF}\frac{\left[ CO_{2} \right]_{IVF}-\left[ CO_{2} \right]_{IVF}^{*}}{\Delta t}=\dot{m}_{CO_{2},f}+\dot{m}_{CO_{2},s}-\dot{m}_{CO_{2},l}+\dot{m}_{CO_{2},ppl}-\dot{m}_{CO_{2},plt}$ | (14) |
| --- | --- | --- |
|  | $\dot{m}_{CO_{2},f}+\dot{m}_{CO_{2},s}=V_{IVF}\frac{\left[ CO_{2} \right]_{IVF}-\left[ CO_{2} \right]_{IVF}^{*}}{\Delta t}+\dot{m}_{CO_{2},l}-\dot{m}_{CO_{2},ppl}+\dot{m}_{CO_{2},plt}$ | (15) |
|  | $\Delta\dot{m}_{CO_{2}}=\dot{m}_{CO_{2},f}+\dot{m}_{CO_{2},s}$ | (16) |

The calculation made use of the assumed farm air volume, $V_{IVF}=4.01\times4.81\times3.01=58.06 m^{3}$, calculated making use of the measured dimensions of the plant farm walls. As CO_2_ imbalance could exist, two values for the CO_2_ concentration were considered: $\left[ CO_{2} \right]_{IVF}$ and $\left[ CO_{2} \right]_{IVF}^{*}$, which are the *desired* CO_2_ concentration in the IVF air, and the *actual* CO_2_ concentration in the plant farm air, respectively ($\mathrm{kg}m^{-3}$). The quantity $\dot{m}_{CO_{2},plt}$ is the mass flowrate of CO_2_ traded between the plants and the IVF air as calculated through the plant model, and $\dot{m}_{CO_{2},ppl}=1.39\times{10}^{-5}\mathrm{kg}s^{-1}$ is the CO_2_ mass rate released by the human worker breath ^14^. The CO_2_ imbalance $\Delta\dot{m}_{CO_{2}}$ was calculated as presented in Equation (16), which was corrected by CO_2_ supply from the air supplied through the fan $\dot{m}_{CO_{2},f}$, or CO_2_ supplied by the cylinder $\dot{m}_{CO_{2},s}$. The time step considered in the plant model was of $15 min=900 s$, equaling the sampling frequency of the sensors in the farm. The variable $\dot{m}_{CO_{2},l}$ is the mass flowrate of CO_2_ that escaped the plant farm through leakages ($\mathrm{kg}s^{-1}$), calculated as presented below in Equation (17),

|  | $\dot{m}_{CO_{2},l}=\frac{\dot{m}_{l}}{\rho_{air}}\left[ CO_{2} \right]_{IVF},$ | (17) |
| --- | --- | --- |

whereas $\dot{m}_{CO_{2},f}$ was calculated as presented Equation (18).

|  | $\dot{m}_{CO_{2},f}=\frac{\dot{m}_{f}}{\rho_{air}}\left[ CO_{2} \right]_{ba},$ | (18) |
| --- | --- | --- |

where $\left[ CO_{2} \right]_{ba}$ is the CO_2_ concentration in the basement supply air ($\mathrm{kg}m^{-3}$).

The CO_2_ imbalance at a given instance in time $\Delta\dot{m}_{CO_{2}}$ was corrected as presented in the algorithm of Supplementary Figure S2. For a given instance in time, the algorithm checked if the plant farm needed CO_2_ supply ($\Delta\dot{m}_{CO_{2}}>0$), removal ($\Delta\dot{m}_{CO_{2}}<0$), or if no correction was necessary ($\Delta\dot{m}_{CO_{2}}=0$), respectively Scenarios A, B and C. After that, a second check compared the CO_2_ concentrations of the supply air and of the plant farm to see if air supply would wash away CO_2_ in the farm ($\left[ CO_{2} \right]_{out}<\left[ CO_{2} \right]_{IVF}^{*}$) or enrich the plant farm atmosphere ($\left[ CO_{2} \right]_{out}>\left[ CO_{2} \right]_{IVF}^{*}$), respectively Scenarios 1 and 2. Depending on the scenarios, the farm made use of the supply air or CO_2_ supply from the cylinders.

When in Scenarios A, the CO_2_ imbalance is corrected by supplying CO_2_ to the plant farm. In the case the CO_2_ concentration of the supply air is smaller than the farm’s (Scenario A.1), supply air would remove CO_2_ from the farm when enriching is needed. As such, air supply is kept to a minimum (i.e., equal to the leakage rate), and the CO_2_ imbalance is corrected by CO_2_ supplied by the cylinder. In the case the CO_2_ concentration of the supply air is larger than the plant farm’s (Scenario A.2), supply air is increased to enrich the atmosphere, and as such, promote the imbalance correction. If the air fan flowrate reaches its maximum value and is not pumping enough CO_2_ into the farm, the rest of the correction is promoted by CO_2_ supply from the cylinders.

When in Scenarios B, the CO_2_ imbalance is corrected by removing CO_2_ from the plant farm, and as such, no CO_2_ is supplied by the cylinder. In the case the CO_2_ concentration of the supply air is smaller than the farm’s (Scenario B.1), supply air would remove CO_2_ from the farm, promoting the imbalance correction. As such, air supply is increased. In the case the CO_2_ concentration of the supply air is larger than the plant farm’s (Scenario B.2), supply air is kept to a minimum (i.e., equal to the leakage rate).

Finally, in Scenario C, there is no imbalance of CO_2_ in the plant farm air. As such, air supply is kept to a minimum (i.e., equal to the leakage rate). In the instance leakage removes CO_2_ from the plant farm, that difference is corrected by supplying CO_2_ from the cylinder. If leakage introduces CO_2_ into the plant farm, no supply comes from the cylinder.

In some cases, CO_2_ imbalance could not be corrected, either because not enough CO_2_ could be extracted from the farm (Scenario B.1 when maximum fan flowrate is reached), or because too much CO_2_ entered the farm through leakages (such as in Scenarios A.2, B.2 and C). This led to an increase in the *actual* IVF air CO_2_ concentration, $\left[ CO_{2} \right]_{IVF}^{*}$, as calculated in Equation (19), to be corrected in the calculation of the next instance in time.

|  | $\left[ CO_{2} \right]_{IVF}^{*}=\frac{\Delta t}{V_{IVF}} \left( \dot{m}_{CO_{2},f}-\dot{m}_{CO_{2},l}+\dot{m}_{CO_{2},s}+\dot{m}_{CO_{2},ppl}-\dot{m}_{CO_{2},plt} \right)+\left[ CO_{2} \right]_{IVF}^{**},$ | (19) |
| --- | --- | --- |

where $\left[ CO_{2} \right]_{IVF}^{**}$ is the *actual* CO_2_ concentration in the IVF air in the previous time instance ($\mathrm{kg}m^{-3}$).

| 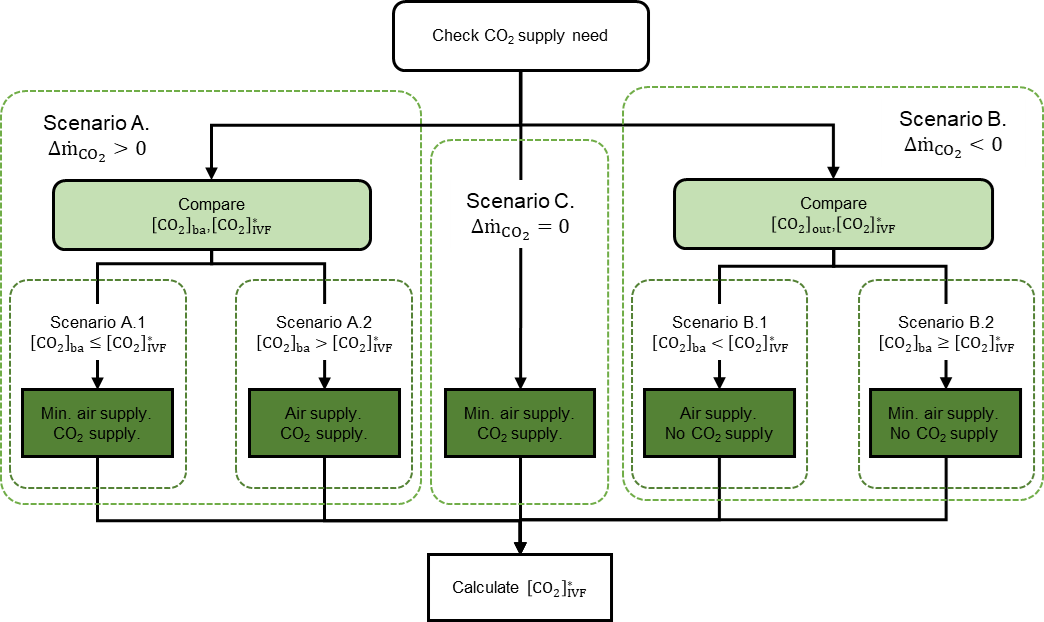 |
| --- |
| Supplementary Figure S2: Flow chart on the CO_2_ control in the plant farm, where $\left[ CO_{2} \right]_{ba}$ and $\left[ CO_{2} \right]_{IVF}^{*}$ are the CO_2_ concentrations of the air supplied and the *actual* concentration of the air inside the IVF, respectively; and $\Delta\dot{m}_{CO_{2}}$ is the CO_2_ imbalance. |

S.1.4.3. Moisture mass balance

Water mass balance was calculated as presented in Equation (20).

|  | $\dot{m}_{w,f}-\dot{m}_{w,l}+\dot{m}_{w,s}-\dot{m}_{w,cond}+\dot{m}_{w,ppl}+\dot{m}_{w,plt,t}=0,$ | (20) |
| --- | --- | --- |
|  | $\dot{m}_{w,s}-\dot{m}_{w,cond}=-\left( \dot{m}_{w,f}-\dot{m}_{w,l}+\dot{m}_{w,ppl}+\dot{m}_{w,plt} \right),$ | (21) |

where $\dot{m}_{w,l}$ is the mass flowrate of moisture leaking out ($\mathrm{kg}s^{-1}$); $\dot{m}_{w,s}$ is the mass flowrate of water supplied by the humidifiers ($\mathrm{kg}s^{-1}$); $\dot{m}_{w,cond}$ is the mass flowrate of water condensed by the air handling unit (AHU) in the IVF ($\mathrm{kg}s^{-1}$); $\dot{m}_{w,ppl}=7.27\times{10}^{-5}\mathrm{kg}s^{-1}$ is the sum of moisture released by the human worker by sweating, $247 g h^{-1}$ ^[15]^ and breathing, $14.78 mL h^{-1}$ ^[16]^; $\dot{m}_{w,plt,t}$ is the moisture released by the plants ($\mathrm{kg}s^{-1})$; and $\dot{m}_{w,f}$ is the mass flowrate of moisture in the supply air ($\mathrm{kg}s^{-1}$), calculated as presented in Equation (22).

|  | $\dot{m}_{w,f}=\frac{\dot{m}_{f}}{\rho_{air}}\left[ H_{2}O \right]_{ba},$ | (22) |
| --- | --- | --- |

where $\left[ H_{2}O \right]_{ba}$ and $\left[ H_{2}O \right]_{IVF}$ are the concentration of water in the supply and IVF air, respectively ($\mathrm{kg}m^{-3}$). Humidity escaping through leakages, $\dot{m}_{w,l}$, was calculated as presented in Equation (23).

|  | $\dot{m}_{w,l}=\frac{\dot{m}_{l}}{\rho_{air}} \left[ H_{2}O \right]_{IVF},$ | (23) |
| --- | --- | --- |

where $\left[ H_{2}O \right]_{ba}$ is the concentration of water in the basement air ($\mathrm{kg}m^{-3}$).

It was assumed that water could not be supplied by the humidifier simultaneously to water being condensed at the heat pump. As such, if the right-hand side of Equation (21) yielded a positive value, moisture was supplied by the humidifier. If it yielded a negative value, moisture was condensed by the heat pump.

S.1.4.4. Energy balance

At this stage, all mass flows were known, being possible to apply an energy balance to the IVF air, presented in Equations (24) and (25), to find if heat needed to be removed from or supplied.

| $q_{f}+q_{l}+ \dot{m}_{w,s}\left( T_{ba} c_{p,l}-h_{w,fg}(T_{ba}) \right)-\dot{m}_{w,cond}\left( T_{IVF} c_{p,v} \right)+c_{p,CO_{2}} \left( \dot{m}_{CO_{2},s} T_{ba} \right)+q_{h}-q_{c}+q_{walls}+q_{ppl}-q_{plt}+q_{equip}=0$ | (24) |
| --- | --- |
| $q_{h}-q_{c}=-\left( q_{f}+q_{l}+ \dot{m}_{w,s}\left( T_{ba} c_{p,l}-h_{w,fg}(T_{ba}) \right)-\dot{m}_{w,cond}\left( T_{IVF} c_{p,v} \right)+c_{p,CO_{2}} \left( \dot{m}_{CO_{2},s} T_{ba} \right)+q_{walls}+q_{ppl}-q_{plt}+q_{equip} \right)$ | (25) |

The constants $c_{p,v}=1862 J kg^{-1} K^{-1}$, $c_{p,l}=4160 J kg^{-1} K^{-1}$ and $c_{p,CO_{2}}=836 J kg^{-1} K^{-1}$ ^17^ are the specific heat at constant pressure of air, water vapor, liquid water and gaseous CO_2_; $h_{w,fg}$ is the enthalpy of vaporization of water calculated for a given temperature ($J kg^{-1}$); $T_{IVF}$ and $T_{ba}$ are the temperatures of the IVF air and of the supply basement air, respectively ($℃$); $q_{l}$ and $q_{walls}$ are the heat removed from the IVF air through leakages and conducted through the farm’s walls, respectively ($W$); $q_{ppl}=197.2 W$ is the heat released by the worker from light work due to metabolic activity ($2 MET$) for an assumed average surface body area of $1.7 m^{2}$ ^18^; $q_{equip}$ is the heat released by the plant farm equipment ($W$); $q_{plt}$ is the heat absorbed by plants during evapotranspiration ($W$); $q_{h}$ and $q_{c}$ are the heat to be supplied and removed from the air by the AHU, respectively ($W$); and $q_{f}$ is the heat entering with the supply air ($W$), as calculated in Equation (26).

| $q_{f}=c_{p,air} \dot{m}_{f} T_{ba}\left( 1-\frac{\left[ H_{2}O \right]_{ba}}{\rho_{air}} \right)+c_{p,v} \dot{m}_{f} T_{ba} \frac{\left[ H_{2}O \right]_{ba}}{\rho_{air}},$ | (26) |
| --- | --- |

where $c_{p,air}=1006{J kg}^{-1} K^{-1}$ is the specific heat at constant pressure of air ^17^.

The heat removed through the leakages was calculated as presented in Equation (27).

| $q_{l}=c_{p,air} \dot{m}_{l} T_{IVF} \left( 1-\frac{\left[ H_{2}O \right]_{IVF}}{\rho_{air}} \right)+c_{p,v} \dot{m}_{f} T_{IVF} \frac{\left[ H_{2}O \right]_{IVF}}{\rho_{air}}$ | (27) |
| --- | --- |

The heat through the walls, $q_{walls}$ ($W$), was calculated as presented below in Equations $(28)$ and $(29)$.

|  | $q_{walls}=\sum_{i=1}^{4} q_{wall,i},$ | $( SEQ Eq. \backslash* ARABIC 28)$ |
| --- | --- | --- |
|  | $q_{wall,i}=\frac{T_{ba}-T_{IVF}}{\frac{2 d_{cl}}{k_{cl} A_{i}}+\frac{d_{f}}{k_{f} A_{i}}+\frac{2}{h_{air} A_{wall, i}}},$ | $( SEQ Eq. \backslash* ARABIC 29)$ |

where $i$ denotes each wall of the plant farm; $A_{wall,i}$ is the area of wall $i$, measuring either $12.07$ or $14.48 m^{2}$; $d_{cl}=0.002 m$ and $d_{f}=0.075 m$ are the thicknesses of the two layers of aluminum cladding and of the one layer of foam composing the walls, respectively; $k_{cl}=239 W m^{-1} K^{-1}$ and $k_{f}=0.03 W m^{-1} K^{-1}$ ^[19,20]^ are the thermal conductivities of the layers of aluminum cladding and foam, respectively; and $h_{air}=2 W m^{-2} K^{-1}$ is the heat transfer coefficient of the air surrounding the walls, estimated for an interior and exterior temperature of $18$ and $24 ℃$, using air properties published in the literature and Churchill and Chu’s correlation for turbulent flow over vertical plates ^21^. The heat absorbed due to the evapotranspiration of the plants, $q_{plt}$, was calculated as presented in Equation $(30)$.

|  | $q_{plt}=\dot{m}_{w,plt,t} h_{fg},$ | $( SEQ Eq. \backslash* ARABIC 30)$ |
| --- | --- | --- |
|  | $h_{fg}=\left( -2.379 T_{PF}+2501 \right)\times{10}^{3},$ | $( SEQ Eq. \backslash* ARABIC 31)$ |

where $h_{fg}$ is the specific enthalpy of vaporization of water ($J kg^{-1}$) for a given temperature ($℃$) calculated as a regression line from data presented online ^17^.

The heat released by the equipment used, $q_{equip}$, is the sum of the heat released by the individual components at the plant farm when operating. Equipment with variable operating schedules is presented in Supplementary Table S1. Photoperiod, and thus the heat emitted by the lamps, was assumed to start at 07:00, and its length varied between 8 to 24 hours of light per day, depending on the photoperiod considered. The basement fan was assumed to always be on, whereas the solenoid valve and the humidifiers released heat as they were used to supply CO_2_ or moisture, as found through the respective mass balances.

The daily consumption of other electric equipment in the farm was of $30872 W h$, and it was assumed half of the supplied energy ($643.2 W$) was dissipated as heat throughout the day, due to lack of knowledge on the efficiency of every single device. The time effects of heat release as radiation were ignored, and as such, the total cooling load at a given moment was assumed to be equal to the total heat gain at that same moment. The same assumption could be found in Van Henten’s original work ^4^.

| Supplementary Table S1. Heat released by equipment at the plant farm. | | | |
| --- | --- | --- | --- |
| Source | Power supplied ($W$) | Conversion to heat | Heat released ($W)$ |
| Lamps | $35\times160$ | $100\%$^a)^ | $5,600$ |
| Solenoid valve (CO_2_ supply) | $8$^b)^ | $100\%$ ^a)^ | $8$ |
| Humidifiers | $230$^c)^$\times2$ | $30\%$^c)^ | $138$ |
| Basement fan | $30$ | $68\%$^d)^ | $20.4$ |
| *Notes*.  ^a)^ Conservative values were used.  ^b)^ MD11-MD13 model sold by Isma ^22^.  ^c)^ Humidifiers of the humiDisk65 model with a consumption of $0.23 kW$ ^23^ and an assumed efficiency of $70\%$, based on efficiencies of electric motors ^24^.  ^d)^ Assumed same efficiency as the larger fans, model FN040-VDK.0F.V7P1, medium settings ^25^. | | | |

When atomized mist was provided to the plant farm from the humidifier, it was assumed it was energy being added to the IVF air, to which was subtracted the energy to evaporate that water. On the other hand, the removed water vapor at the condenser, $\dot{m}_{w,cond}$, removed with it energy from the IVF air. The cooling power required to cool down and condensate the excess moisture, presented in Equation $(32)$, was provided by the AHU, and as such did not enter in the plant farm energy balance.

|  | $q_{cond}=\dot{m}_{w,cond} \left( c_{p,v} \left( T_{cond}-T_{IVF} \right)-h_{fg}\left( T_{cond} \right) \right),$ | $( SEQ Eq. \backslash* ARABIC 32)$ |
| --- | --- | --- |

where $T_{cond}=5 ℃$ is the assumed AHU condenser temperature.

As done before for the supply/removal of CO_2_, $q_{h}$ and $q_{c}$ cannot be non-zero simultaneously. The electricity consumption of the AHU, $E_{AHU}$ ($W$), was then calculated as presented in Equation $(33)$.

|  | $E_{AHU}=\frac{q_{h}}{COP_{h}}-\frac{q_{c}+q_{cond}}{COP_{c}},$ | $( SEQ Eq. \backslash* ARABIC 33)$ |
| --- | --- | --- |

where $COP_{h}=1$ and $COP_{c}=3$ are the coefficients of performance of the heating resistance and of the cooling heat pump, respectively ^26^.

To the amount of CO_2_ calculated during plant farm operation was added the mass of CO_2_ necessary to bring the CO_2_ concentration in the plant farm during the setting up stage from ambient concentration to $\left[ CO_{2} \right]_{IVF}$. For conditioning the room, it was considered a CO_2_ release rate from the cylinder of $1 g s^{-1}$, with a constant air leakage rate of $0.015 ACH$.

S.1.5. Life cycle inventories

Primary LCI sources for farm material inputs and process specifications for the infrastructure and combination of ag-tech used (growth chamber, LEDs and growing systems) were taken from an article by Parkes et al. ^3^.

S.1.5.1. Energy mix

The energy mix considered in the inventory was replaced from ecoinvent’s process for electricity production in Portugal in 2018 by the average production mix practiced in Portugal between January and August of 2022: $34.1\%$ natural gas, $6.4\%$ fossil CHP, $11.1\%$ hydro, $33.4\%$ wind, $8.3\%$ bioenergy and $6.6\%$ solar ^27^. The inclusion of the electricity transmission network, and input and emission of sulfur hexafluoride were based on the process for electricity supply from the ecoinvent database. The values for non-PV energy vectors consider the $4.4\%$ loss considered in ecoinvent’s database due to conversion from high voltage to low voltage.

| Supplementary Table S2: Life cycle inventory for the electricity energy mix used in Portugal. | | | |
| --- | --- | --- | --- |
| Name | Amount | Unit | Ecoinvent process |
| Inputs | | | |
| Natural gas electricity production | $35.7$ | $\mathrm{kWh}$ | electricity production, natural gas, conventional power plant |
| Fossil CHP electricity production | $6.7$ | $\mathrm{kWh}$ | heat and power co-generation, natural gas, conventional power plant, 100MW electrical |
| Hydro electricity production | $11.6$ | $\mathrm{kWh}$ | electricity production, hydro, reservoir, non-alpine region |
| Wind electricity production | $34.9$ | $\mathrm{kWh}$ | electricity production, wind, 1-3MW turbine, onshore \| electricity, high voltage; electricity production, hydro, pumped storage \| electricity, high voltage |
| Bioenergy electricity production | $8.7$ | $\mathrm{kWh}$ | heat and power co-generation, wood chips, 6667 kW, state-of-the-art 2014 |
| Photovoltaic electricity production | $6.6$ | $\mathrm{kWh}$ | electricity production, photovoltaic, 570kWp open ground installation, multi-Si |
| Distribution network | $8.74\times{10}^{-6}$ | $\mathrm{km}$ | distribution network, electricity, low voltage |
| Liquid sulfur hexafluoride | $6.27\times{10}^{-7}$ | $\mathrm{kg}$ | sulfur hexafluoride, liquid |
| Outputs | | | |
| Low voltage electricity (PT) | $100$ | $\mathrm{kWh}$ | Electricity, in Portugal |
| Sulfur hexafluoride emission to air | $6.27\times{10}^{-7}$ | $\mathrm{kg}$ | Sulfur hexafluoride |

S.1.5.2. CO_2_ cylinders

Added to the inventory were two CO_2_ aluminum cylinders, used to increase the farm air CO_2_ concentration to promote the plant growth: when the first cylinder is emptied, it is replaced by the second while the farm staff get it refilled. It is assumed that the cylinders are manufactured in Nottingham, England by Luxfer ^28^, transported by 16-ton freight truck to London Gateway ($245 \mathrm{km}$), by ship freight from London Gateway to Bilbao, Spain ($1257 km$), and by truck freight from Bilbao to Vila-seca ($531 km$), where they are filled with liquid CO_2_ ^29^. They are then transported to the distribution center in Lisbon ^30^ by freight truck ($1119 km$), where the customer buys the bottle and drives to the campus ($28.9 km$), assumed in a light vehicle. For every cylinder refill necessary, transport from Carcavelos to Vila-seca and back is necessary through the same means.

The cylinder is based in the model L3239 from the supplier’s website ^28^ that weighs $25.5 kg$ and can transport $26.7 kg$ at $100\%$ capacity. The cylinder can reach a lifetime of $30$ years ^31^. The main elements of the gas cylinder considered is the aluminum cylinder itself, the valve of the cylinder, and the plastic handle and turning wheel. The material of the cylinder’s surface considered is 6061-T6 high strength alloy aluminum, which is modeled as generic aluminum wrought alloy and the energy required in metal working $797 kWh kg^{-1}$ ^[32,33]^. The valve is stated to be according to the model CGA320, which was assumed to weigh $2 lb$ ($0.91 \mathrm{kg}$) and be made of brass through forging^34,35^ and machined (assumed $15\%$ material loss), modelled in Ecoinvent as forging and machining for aluminum, as this is the metal with the closest ultimate tensile strength to brass. The handle of the cylinder is stated to be made of nylon^36^, so it was assumed to be made of nylon 6-6 and shaped with injection molding. The same was assumed for the turning wheel of the valve. It was assumed both nylon items weighted in conjunction $0.3 \mathrm{kg}$. As to end-of-life, the cylinder is assumed to be disassembled: metals are recycled, and plastics are treated as municipal solid waste. The process for disposal of brass is modelled by the process for copper due to limitations in the database. The inventory for the cylinder is presented below.

| Supplementary Table S3: Life cycle inventory for the CO_2_ cylinder. | | | | |
| --- | --- | --- | --- | --- |
| **Name** | | **Amount** | | **Ecoinvent process** |
| **Inputs** | | | | |
| Cylinder | Aluminum, wrought alloy | $24.3$ | $\mathrm{kg}$ | aluminum, wrought alloy |
|  | Reverse extrusion | $24.3$ | $\mathrm{kg}$ | section bar extrusion, aluminum |
|  | Heat treatments | $19.4$ | $\mathrm{MWh}$ | electricity, low voltage |
| Valve | Brass | $0.907$ | $\mathrm{kg}$ | brass |
|  | Forging | $0.907$ | $\mathrm{kg}$ | impact extrusion of aluminum, 1 stroke |
|  | Machining | $0.136$ | $\mathrm{kg}$ | brass removed by drilling, computer numerical controlled |
| Handle and  wheel | Injection molding (nylon) | $0.3$ | $\mathrm{kg}$ | injection moulding |
|  | Nylon 6-6 | $0.3$ | $\mathrm{kg}$ | nylon 6-6 |
| Transportation | 16-ton truck transport | $79.68$ | $t km$ | transport, freight, lorry 16-32 metric ton, EURO6 |
|  | Container ship | $32.05$ | $t km$ | transport, freight, sea, container ship |
|  | Light vehicle transport | $1.508$ | $t km$ | transport, freight, light commercial vehicle |
| **Outputs** | | | | |
| Aluminum cylinder | | $1$ | $\mathrm{Items}$ | CO_2_ cylinder, in NOVA |
| Aluminum waste | | $24.3$ | $\mathrm{kg}$ | aluminum scrap, post-consumer |
| Brass waste | | $0.907$ | $\mathrm{kg}$ | copper scrap, sorted, pressed |
| Nylon waste | | $0.3$ | $\mathrm{kg}$ | waste plastic, mixture |

S.1.5.3. Flowrate controller

A flowrate controller was also considered to manage the supply of CO_2_ to the air. It is assumed as a solenoid valve, the MD11-MD13 model sold by Isma ^22^, based on Vila Nova da Telha, Portugal, from which is assumed to be directly transported to the farm in a lightweight vehicle ($348 \mathrm{km}$). The body is assumed to be made of $1.0 kg$ of forged brass, $15\%$ of which assumed machined off, whereas the solenoid valve is assumed to be comprised of $0.2 kg$ of nylon 6‑6 for the casing, $0.1 kg$ of copper wire for the coil, and $0.2 kg$ of stainless steel for the core and inner components. It is assumed the valve has a lifetime of $3$ years, at the end of which metallic parts are recycled and plastic parts are treated as municipal solid waste.

| Supplementary Table S4: Life cycle inventory for the CO_2_ flowrate controller. | | | | | | | | |
| --- | --- | --- | --- | --- | --- | --- | --- | --- |
| Name | | Amount | | | | | | Ecoinvent process |
| Inputs | | | | | | | | |
| Body | Brass | | $1$ | | $\mathrm{kg}$ | | brass | |
|  | Forging | | $1$ | | $\mathrm{kg}$ | | impact extrusion of aluminum, 1 stroke | |
|  | Machining | | $0.15$ | | $\mathrm{kg}$ | | brass removed by drilling, computer numerical controlled | |
| Solenoid valve | Nylon 6-6 | | $0.2$ | | $\mathrm{kg}$ | | nylon 6-6 | |
|  | Injection molding | | $0.2$ | | $\mathrm{kg}$ | | injection moulding | |
|  | Stainless steel | | $0.2$ | | $\mathrm{kg}$ | | steel, chromium steel 18/8 | |
|  | Extrusion | | $0.2$ | | $\mathrm{kg}$ | | impact extrusion of steel, cold, 1 strokes | |
|  | Copper | | $0.1$ | | $\mathrm{kg}$ | | copper, cathode | |
|  | Wire drawing | | $0.1$ | | $\mathrm{kg}$ | | wire drawing, copper | |
| Transport | Light vehicle transport | | $522$ | | $kg km$ | | transport, freight, light commercial vehicle | |
| Outputs | | | | | | | | |
| CO_2_ flowrate controller | | $1$ | | $\mathrm{Items}$ | | CO_2_ flowrate controller, in NOVA | | |
| Brass waste | | $1$ | | $\mathrm{kg}$ | | copper scrap, sorted, pressed | | |
| Nylon waste | | $0.2$ | | $\mathrm{kg}$ | | waste plastic, mixture | | |
| Steel waste | | $0.2$ | | $\mathrm{kg}$ | | waste bulk iron, excluding reinforcement | | |
| Copper waste | | $0.1$ | | $\mathrm{kg}$ | | copper scrap, sorted, pressed | | |

S.1.5.4. Supplying CO_2_

The quantities in Supplementary Table S5 are given by the Equations $(34)$ to $(36)$ and below.

|  | $n_{Ref}=\frac{m_{CO_{2},s}}{26.7},$ | $( SEQ Eq. \backslash* ARABIC 34)$ |
| --- | --- | --- |
|  | $T_{car}=\left( 28.9 km \right)\times\left( 25.5 kg+\left( 25.5+26.7 \right) kg \right) n_{Ref}=2244.57 n_{Ref},$ | $( SEQ Eq. \backslash* ARABIC 35)$ |
|  | $T_{trk}=\left( 1119 km \right)\times\left( 25.5 kg+\left( 25.5+26.7 \right) kg \right) n_{Ref}=86909 n_{Ref},$ | $( SEQ Eq. \backslash* ARABIC 36)$ |

where $n_{Ref}$ is the number of cylinder refills per week; $m_{CO_{2},s}$ is the mass of CO_2_ supplied to the plant farm per week ($\mathrm{kg}$); $T_{car}$ is the necessary transport by light vehicle due to refilling ($kg km$); and $T_{trk}$ is the necessary transport by 16-ton freight truck due to refilling ($kg km$).

| Supplementary Table S5: Life cycle inventory for the supply of CO_2_ to the plant farm’s air. | | | | |
| --- | --- | --- | --- | --- |
| Name | | Amount | | Ecoinvent process |
| Inputs | | | | |
| Liquid CO_2_ | | $m_{\mathrm{CO}_{2}}$ | $\mathrm{kg}$ | carbon dioxide, liquid |
| Refilling | Transport by light vehicle | $T_{\mathrm{car}}$ | $kg km$ | transport, freight, light commercial vehicle |
|  | Transport by 16-ton freight truck | $T_{\mathrm{trk}}$ | $kg km$ | transport, freight, lorry 16-32 metric ton, EURO6 |
| Output | | | | |
| CO_2_ supply | | $m_{\mathrm{CO}_{2},s}$ | $\mathrm{kg}$ | CO_2_ supply, in NOVA |

The amount $m_{CO_{2},e}$ is the part of CO_2_ excess that is ventilated out of the plant farm being supplied from the cylinder. If all CO_2_ supplied to the plants sourced from the building, $m_{CO_{2},e}=0$, as all CO_2_ released by the plants originated in CO_2_ otherwise released by the building into the atmosphere, thus being a burden-free in the point of view of the farm. However, if part of the CO_2_ absorbed by the plants is sourced from the cylinder, a portion of the CO_2_ released by the plants includes the GWP associated with the release of CO_2_e from bottled CO_2_. As such, $m_{CO_{2},e}$ was calculated as the total CO_2_ mass released by the farm multiplied by the CO_2_ ratio supplied by the cylinder to total CO_2_ supplied to the plants.

S.1.5.5. Humidifiers

Another type of item added to the plant farm’s inventory is the humidifier. The two humidifiers present in the plant farm were considered as the CarelUC humiDisk65 ^37^ (Michael Parkes pers. com., May 2022), whose spare parts website showcases information on some of the parts of the device ^38^. The user manual states that the device weighs a total of $17.6 \mathrm{kg}$ ^23^. It contains an electric motor ($9.19 kg$), and its inventory and disposal were approximated by the one of an electric scooter motor in the ecoinvent database. The disposal of $1 kg$ of this motor results in waste treatment of $0.3296 kg$ of aluminum, $0.1398 kg$ of copper, $0.0549 kg$ of iron, $0.0299 kg$ of polyethylene and $0.5617 kg$ of steel, excluding waste generated during manufacture, already accounted within the process. The humidifier also includes a solenoid valve ($0.26 kg$), whose inventory was assumed the same as the one presented in Supplementary Table S4. The humidifier also contains an air filter ($0.05 kg$), assumed to be made of polyurethane foam; temperature sensors; a level controller ($0.07 kg$); and an electronic card ($0.50 kg$). The rest of the weight of the device ($7.48 kg$) was assumed as structural or mechanical components made of ABS. A wall support ($0.96 kg$) modelled after the one for humiDisk10 ^39^ is also required. The hoses considered are a $0.35 kg$ plastic hose ^38^ and $0.55 kg$ braided steel hose, also modelled after humiDisk10’s ^39^.The units for the level controller had to be converted from a weight basis to length using the conversion factor presented in ecoinvent ($0.065 kg m^{-1}$).

The total weight of the equipment and accessories is assumed as $19.46 kg$. It is assumed that a humidifier lasts $15$ years, then being disassembled: metal parts are recycled, and plastic parts treated as municipal solid waste. The humidifier has a supply power of $0.23 kW$. Transportation from the manufacturer in Labin, Croatia ^40^ to Lisbon is assumed to happen by freight truck ($2621 km$), and from the assumed distributor Pecomark facility in Portugal ^41^ to the NOVA campus ($39 km$) by light vehicle.

| Supplementary Table S6: Life cycle inventory for the air humidifiers used | | | | |
| --- | --- | --- | --- | --- |
| Name | | Amount | | Ecoinvent process |
| Inputs | | | | |
| Electric motor | | $9.19$ | $\mathrm{kg}$ | electric motor production, for electric scooter |
| Solenoid valve | Nylon 6-6 | $0.104$ | $\mathrm{kg}$ | nylon 6-6 |
|  | Injection molding | $0.104$ | $\mathrm{kg}$ | injection moulding |
|  | Stainless steel | $0.104$ | $\mathrm{kg}$ | steel, chromium steel 18/8 |
|  | Extrusion | $0.104$ | $\mathrm{kg}$ | impact extrusion of steel, cold, 1 strokes |
|  | Copper | $0.052$ | $\mathrm{kg}$ | copper, cathode |
|  | Wire drawing | $0.052$ | $\mathrm{kg}$ | wire drawing, copper |
| Level controller | | $1.077$ | $m$ | cable production, connector for computer, without plugs |
| Air filter | | $0.05$ | $\mathrm{kg}$ | polyurethane, flexible foam |
| Sensors | | $2$ | $\mathrm{Items}$ | Gh Sensors, from the original inventory ^3^ |
| Electronic card | | $0.5$ | $\mathrm{kg}$ | electronics, for control units |
| Structure | ABS | $7.48$ | $\mathrm{kg}$ | acrylonitrile-butadiene-styrene copolymer |
|  | Injection molding | $7.48$ | $\mathrm{kg}$ | injection moulding |
| Wall support | Stainless steel | $0.98$ | $\mathrm{kg}$ | steel production, chromium steel 18/8, hot rolled |
|  | Drilling | $0.02$ | $\mathrm{kg}$ | chromium steel drilling, computer numerical controlled |
| Plastic hose | PVC | $0.35$ | $\mathrm{kg}$ | polyvinylidenchloride production, granulate |
|  | Extrusion | $0.35$ | $\mathrm{kg}$ | extrusion, plastic pipes |
| Steel hose | Stainless steel | $0.55$ | $\mathrm{kg}$ | steel, chromium steel 18/8 |
|  | Braiding | $0.55$ | $\mathrm{kg}$ | metal working, average for steel product manufacture |
| Transportation | Transport by 16-ton truck | $51.00$ | $t km$ | transport, freight, lorry 16-32 metric ton, EURO6 |
|  | Transport by light vehicle | $758.94$ | $kg km$ | transport, freight, light commercial vehicle |
| Outputs | | | | |
| Humidifier | | $1$ | $\mathrm{Items}$ | Humidifier, in NOVA |
| steel waste | | $7.281$ | $\mathrm{kg}$ | waste bulk iron, excluding reinforcement |
| aluminum waste | | $3.029$ | $\mathrm{kg}$ | aluminum scrap, post-consumer |
| copper waste | | $1.337$ | $\mathrm{kg}$ | copper scrap, sorted, pressed |
| polyethylene waste | | $0.2748$ | $\mathrm{kg}$ | waste plastic, mixture |
| ABS waste | | $7.48$ | $\mathrm{kg}$ | waste plastic, mixture |
| Nylon 6-6 waste | | $0.104$ | $\mathrm{kg}$ | waste plastic, mixture |
| PVC waste | | $0.35$ | $\mathrm{kg}$ | waste polyvinylchloride |
| Polyurethane waste | | $0.05$ | $\mathrm{kg}$ | waste polyurethane |
| waste level controller | | $0.07$ | $\mathrm{kg}$ | used cable |
| Electronic card waste | | $0.5$ | $\mathrm{kg}$ | electronics scrap from control units |

S.1.5.6. Indoor vertical farm operation

The IVF operation is represented in Supplementary Table S7, and includes the equipment and the mass and energy flows required for the weekly production of kale.

The first four items in Supplementary Table S7 are directly pulled from the original inventory ^3^, and as such are not presented in this work. The authors had used data for cauliflower seeds from the Agribalyse v.3.0.1 database as a model for broccoli seeds. Belonging to the same species as broccoli and cauliflower (*Brassica oleracea*), kale seeds were also assumed to be modelled as cauliflower seeds. The plant farm infrastructure has an assumed lifetime of $20$ years whereas the LED bars are assumed to last $10$ years ^3^.

The constants $n$ represent the required amount of each item per week calculated with the lifetime of each respective item in weeks: $n_{GH}=1/(52\times20)=9.6\times{10}^{-4}$ is the number of plant farm infrastructures; $n_{LEDs}=1/(52\times10)=1.92\times{10}^{-3}$ is the number of LED systems; $n_{cyl}=2/(30\times52)=6.393\times{10}^{-4}$is the number of aluminum cylinders; $n_{f,c}=1/(3\times52)=6.393\times{10}^{-3}$ is the number of CO_2_ flowrate controllers; and $n_{h}=2/(52\times15)=2.557\times{10}^{-3}$ is the number of humidifiers. The total amount of CO_2_ supply required, $m_{CO_{2},s}$, is obtained through the methodology explained above, whereas the total amount of water required, $m_{w,T}$, is the sum of the mist supplied to the plant farm, $m_{w,s}$, and the water needed for the plants $m_{w,plt,c}$, as calculated through Equation $(7)$. The total electricity required, $E_{T}$, is calculated as the sum of energy spent on the small fan ($30 W$), on the $160$ LED bars ($35 W$ each), on the solenoid valve of the CO_2_ cylinder ($8 W$), on the two humidifiers ($0.23 kW$ each), and on other plant farm equipment. The masses of fertilizers, $m_{Ca\left( NO_{3} \right)_{2}}$, $m_{NH_{4}H_{2}PO_{4}}$ and $m_{KNO_{3}}$ necessary to allow plant growth are also included. The quantity $m_{FW,T}$ represents the total fresh weight harvested by the end of the week ($\mathrm{kg}$).

| Supplementary Table S7: Life cycle inventory for the weekly production of fresh weight of kale. | | | |
| --- | --- | --- | --- |
| Name | Amount | | Ecoinvent process |
| Inputs | | | |
| Indoor Vertical Farm usage | $n_{GH}$ | $\mathrm{Items}$ | Plant farm, installed |
| Clean material | $1$ | $\mathrm{Items}$ | Clean material |
| LED fixtures | $n_{LEDs}$ | $\mathrm{Items}$ | LED fixtures |
| Seeded trays | $1$ | $\mathrm{Items}$ | Seeded trays |
| Aluminum cylinder | $n_{cyl}$ | $\mathrm{Items}$ | CO_2_ cylinder, in NOVA |
| CO_2_ flowrate controller | $n_{f,c}$ | $\mathrm{Items}$ | CO_2_ flowrate controller, in NOVA |
| Supplied CO_2_ | $m_{CO_{2},s}$ | $\mathrm{kg}$ | CO_2_ supply, in NOVA |
| Humidifier | $n_{h}$ | $\mathrm{Items}$ | Humidifier, in NOVA |
| Water | $m_{w,T}$ | $\mathrm{kg}$ | tap water |
| Electricity | $E^{T}$ | $\mathrm{kWh}$ | Electricity, in Portugal |
| Calcium nitrate | $m_{Ca\left( NO_{3} \right)_{2}}$ | $\mathrm{kg}$ | calcium nitrate |
| Monoammonium phosphate | $m_{NH_{4}H_{2}PO_{4}}$ | $\mathrm{kg}$ | monoammonium phosphate |
| Potassium nitrate | $m_{KNO_{3}}$ | $\mathrm{kg}$ | potassium nitrate |
| Outputs | | | |
| Ready for harvest kale | $m_{FW,T}$ | $\mathrm{kg}$ | Kale, unharvested |
| Excess CO_2_ | $m_{CO_{2},e}$ | $\mathrm{kg}$ | Carbon dioxide |

The amount $m_{CO_{2},e}$ is the part of CO_2_ excess that is ventilated out of the plant farm being supplied from the cylinder. If all CO_2_ supplied to the plants sourced from the building, $m_{CO_{2},e}=0$, as all CO_2_ released by the plants would have origin in CO_2_ that would otherwise be released by the building into the atmosphere, thus being a burden-free release in the point of view of the IVF. However, if part of the CO_2_ absorbed by the plants are sourced from the cylinder, a portion of the CO_2_ released by the plants must include the global warming potential (GWP) associated with the release of CO_2_e from bottled CO_2_. As such, $m_{CO_{2},e}$ is calculated as the total mass of CO_2_ released by the farm multiplied by the mass ratio of CO_2_ supplied by the cylinder to total CO_2_ supplied to the plants.

The processes then follow as shown in the work of Parkes et al. ^3^, with the harvesting of the microgreens, separating shoots from roots, and turning the roots and substrate into compost, whose process is presented in Supplementary Table S8. The quantities considered are calculated as presented below in Equations $(37)$ and $(38)$,

|  | $m_{FW,T,s}=m_{FW,T} \left( 1-c_{\tau} \right),$ | $( SEQ Eq. \backslash* ARABIC 37)$ |
| --- | --- | --- |
|  | $m_{FW,T,r}=m_{FW,T} c_{\tau},$ | $( SEQ Eq. \backslash* ARABIC 38)$ |

where $m_{FW,T,s}$ and $m_{FW,T,r}$ are the total fresh weight of shoots and roots ($\mathrm{kg}$), respectively; and $c_{\tau}$ is the root to total DW ratio as defined by Van Henten ^4^. It is assumed this ratio is also true for the fresh weight quantities of root to total crop mass. The mass of the substrate is given in the original inventory as $3 kg$ per kilogram of kale shoots. As in the article it was assumed a daily yield of $7.5 kg$ of kale shoots, it implies a total of $m_{sub}=157.5 kg$ of substrate was used throughout the week.

| Supplementary Table S8: Life cycle inventory for the weekly treatment of consumable kale shoots. | | | |
| --- | --- | --- | --- |
| Name | Amount | | Ecoinvent process |
| Inputs | | | |
| Ready for harvest kale | $m_{FW,T}$ | $\mathrm{kg}$ | Kale, unharvested |
| Substrate | $m_{sub}$ | $\mathrm{kg}$ | Substrate |
| Treatment equipment | $n_{tr}$ | $\mathrm{Items}$ | Treatment Equipment |
| Outputs | | | |
| Kale shoots | $m_{FW,T,s}$ | $\mathrm{kg}$ | Kale, ready for consumption |
| Compostable biomass | $m_{FW,T,r}+m_{sub}$ | $\mathrm{kg}$ | compost |

The constant $n_{tr}$ is the amount of equipment required throughout the week and is calculated based on a lifetime of $10$ years, $n_{tr}=1/(52\times10)=1.92\times{10}^{-3}$. The environmental impacts calculation is then made for the functional unit of $1 kg$ of kale shoots, ready for consumption. As transportation of crop between farm gate and consumer is done by foot in the same building, no further inputs are considered.

S.2. Results

S.2.1. Calibration results

The calibration of parameters of the plant model, as explained in section S.1.1., resulted in a set of values in the same order of magnitude as the values proposed by Van Henten. As such, they were the values used in the plant model in the rest of the present work. The areas used in the experiments estimated from the calibration were of $A_{1}=0.168 m^{2}$ and $A_{2}=0.0340 m^{2}$, respectively for the works of Ford and Thorne^1^ and Chowdhury et al. ^2^.

| Supplementary Table S9: Values for the parameters for lettuce ^4^ and calibrated for kale. | | | | | | | |
| --- | --- | --- | --- | --- | --- | --- | --- |
| Sets of values | $c_{\beta}$ | $c_{\varepsilon}$($\mathrm{kg}J^{-1}$) | $c_{car,2}$  ($m s^{-1} ^{\circ}C^{-1}$) | $c_{k}$ | $c_{\mathrm{lar}}$  ($m^{2} kg^{-1}$) | $c_{car,1}$($m s^{-1} ℃^{-2}$) | $c_{car,3}$($m s^{-1}$) |
| Lettuce | $0.800$ | $1.70\times{10}^{-8}$ | $5.97\times{10}^{-4}$ | $0.900$ | $62.5$ | $-1.32\times{10}^{-5}$ | $-2.64\times{10}^{-3}$ |
| Kale | $0.757$ | $2.15\times{10}^{-8}$ | $0.0011$ | $0.704$ | $62.6$ | $-2.62\times{10}^{-5}$ | $-9.65\times{10}^{-3}$ |

S.2.2. Electricity supply

| 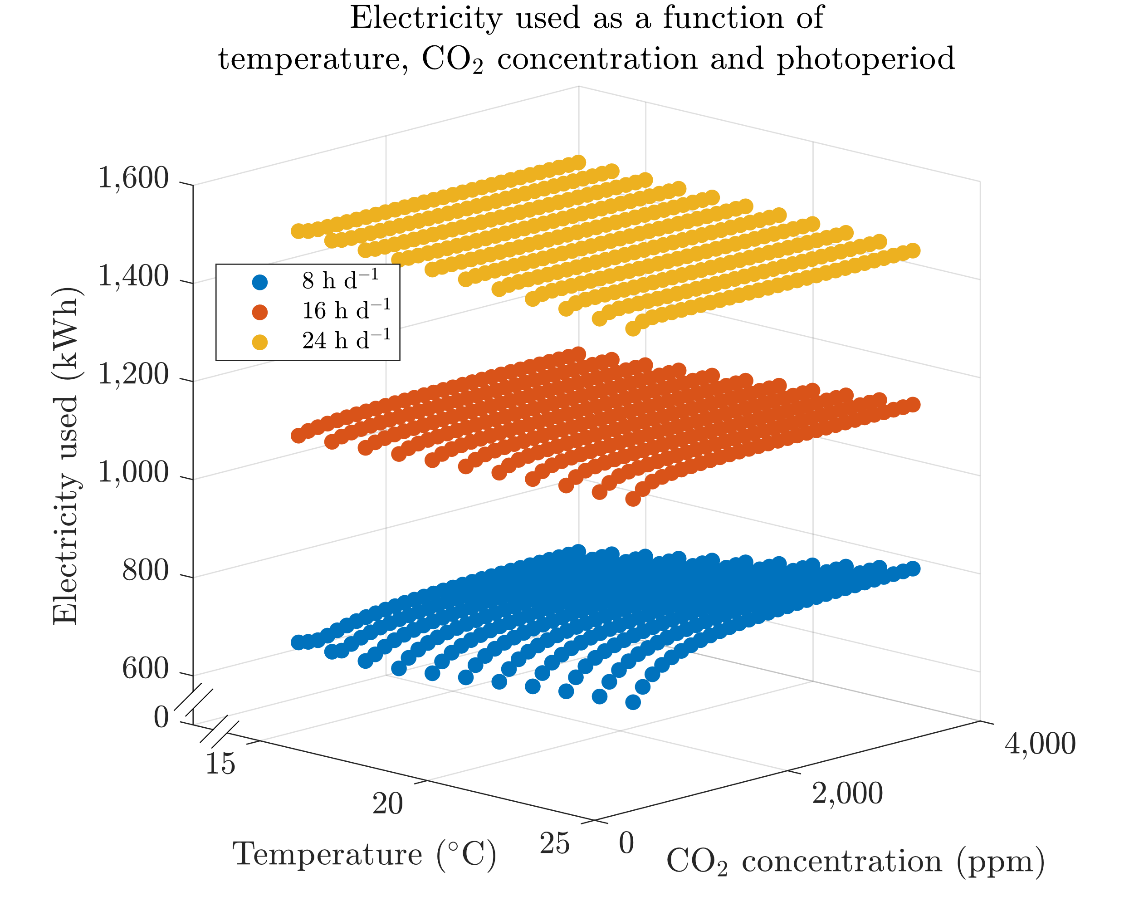 |
| --- |
| Supplementary Figure S3: Electricity supply as a function of temperature, CO_2_ concentration and photoperiod. Only three values for photoperiod are represented for clarity: $8 h d^{-1}$ (blue), $16 h d^{-1}$ (orange) and $24 h d^{-1}$ (yellow). |
| 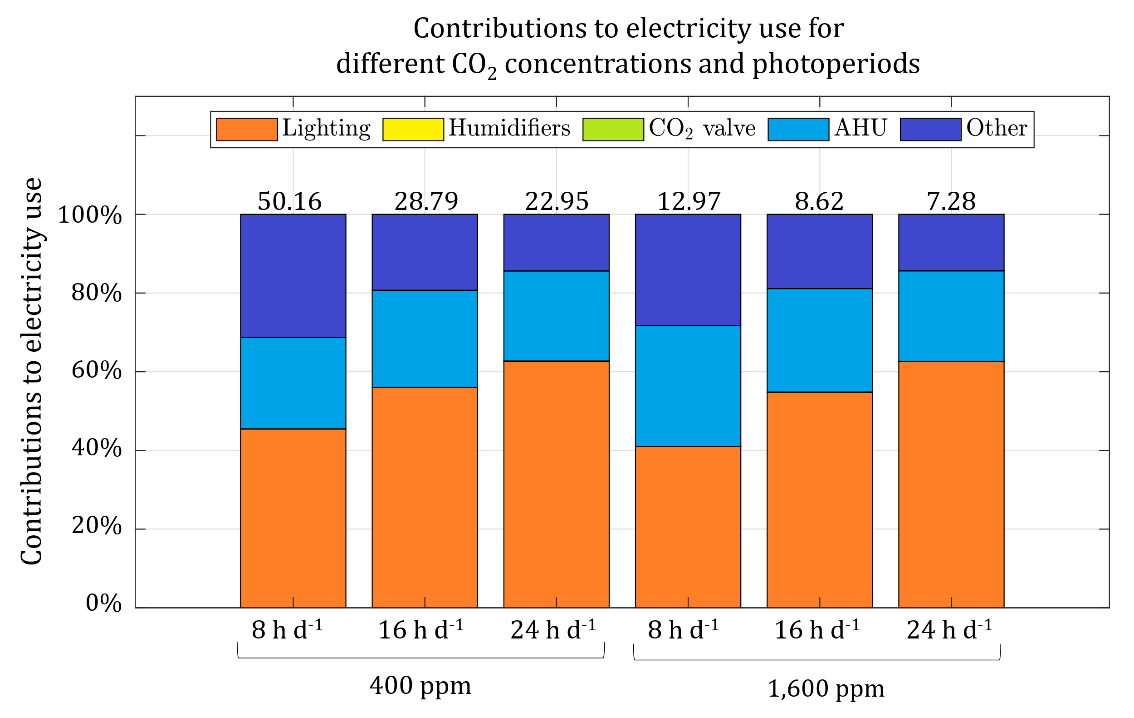 |
| Supplementary Figure S4: Contributions to electricity use per unit weight of harvest ($kWh kg^{-1}$) of the different inputs required for the production of kale in the plant farm for two CO_2_ concentrations, $400$ and $1,600 ppm$, and three photoperiods, $8$, $16$ and $24 h d^{-1}$, and a temperature of $20 ℃$. |

S.2.3. Other impact categories

|  |
| --- |
| Supplementary Figure S5: Electricity supply as a function of temperature, CO_2_ concentration and photoperiod. Only three values for photoperiod are represented for clarity: $8 h d^{-1}$ (blue), $16 h d^{-1}$ (orange) and $24 h d^{-1}$ (yellow). |

Nomenclature

The following table 10 is a list of the variables used throughout this document.

| Supplementary Table S10: Completed list variables used throughout this document and manuscript. |
| --- |

| **Symbol** | **Explanation** | **Units** |
| --- | --- | --- |
| **Indoor vertical farm (IVF) air characteristics** | | |
| $T_{IVF}$ | Temperature of the IVF air | $℃$ |
| $\left[ H_{2}O \right]_{IVF}$ | Water concentration in the IVF air | $\mathrm{kg}m^{-3}$ |
| $\left[ H_{2}O \right]_{sat}$ | Water saturation concentration of the IVF air | $\mathrm{kg}m^{-3}$ |
| $\left[ CO_{2} \right]_{IVF}$ | Desired CO_2_ concentration in the IVF air | $\mathrm{kg}m^{-3}$ |
| $\left[ CO_{2} \right]_{IVF}^{*}$ | Actual CO_2_ concentration in the IVF air in the current time step of calculation | $\mathrm{kg}m^{-3}$ |
| $\left[ CO_{2} \right]_{IVF}^{**}$ | Actual CO_2_ concentration in the IVF air in the previous time step of calculation | $\mathrm{kg}m^{-3}$ |
| **Basement air characteristics** | | |
| $T_{ba}$ | Temperature of the basement air | $℃$ |
| $\left[ H_{2}O \right]_{ba}$ | Water concentration in the basement air | $\mathrm{kg}m^{-3}$ |
| $\left[ CO_{2} \right]_{ba}$ | CO_2_ concentration in the basement air | $\mathrm{kg}m^{-3}$ |
| **Plant model** | | |
| $m_{DW}$ | Plant dry mass | $\mathrm{kg}\mathrm{DW}$ |
| $\phi_{phot}$ | CO_2_ assimilation rate | $kg CO_{2} s^{-1}$ |
| $\phi_{resp}$ | CO_2_ respiration rate | $kg CO_{2} s^{-1}$ |
| $c_{\beta}$ | Yield factor | Adim. |
| $c_{\varepsilon}$ | Light use efficiency at high CO_2_ concentrations | $\mathrm{kg}J^{-1}$ |
| $c_{car,1}$ | Coefficient relating air temperature to carboxylation conductance of CO_2_ through the leaves | $m s^{-1} ℃^{-2}$ |
| $c_{car,2}$ | Coefficient relating air temperature to carboxylation conductance of CO_2_ through the leaves | $m s^{-1} ^{\circ}C^{-1}$ |
| $c_{car,3}$ | Coefficient relating air temperature to carboxylation conductance of CO_2_ through the leaves | $m s^{-1}$ |
| $c_{k}$ | Light extinction coefficient | Adim. |
| $c_{lar}$ | Leaf area ratio | $m^{2} kg^{-1}$ |
| $c_{par}$ | Fraction of photosynthetically active radiation (PAR) | Adim. |
| $c_{par,J}$ | PAR efficiency | $\mu mol J^{-1}$ |
| $V_{i}$ | Light intensity | $W m^{-2}$ |
| $V_{i,PFD}$ | Total photon flux density | $\mu mol m^{-2}s^{-1}$ |
| $c_{rad,rf}$ | Transmission coefficient | Adim. |
| $c_{DW}$ | Dry to fresh weight ratio | Adim. |
| $\dot{m}_{w,plt,t}^{''}$ | Moisture release rate by the plants | $\mathrm{kg}m^{-2} s^{-1}$ |
| $\dot{m}_{w,plt,c}^{''}$ | Water consumption rate by the plants | $\mathrm{kg}m^{-2} s^{-1}$ |
| $c_{pl,d}$ | Effective canopy surface | $m^{2} kg^{-1}$ |
| $m_{DW}^{''}$ | Dry weight per unit area at a given time | $\mathrm{kg}m^{-2}$ |
| $\sigma_{H_{2}O}$ | Water conductance through the leaves | $m s^{-1}$ |
| $c_{stm}^{H_{2}O}$ | Stomatal conductance to water transport through the leaves | $m s^{-1}$ |
| $c_{bnd}^{H_{2}O}$ | Boundary layer conductance to water transport through the leaves | $m s^{-1}$ |
| $c_{stm}^{CO_{2}}$ | Stomatal conductance to CO_2_ transport through the leaves | $m s^{-1}$ |
| $c_{bnd}^{CO_{2}}$ | Boundary layer conductance to CO_2_ transport through the leaves | $m s^{-1}$ |
| $c_{n,N}$ | Nitrogen content in plant dry weight | $g kg DW^{-1}$ |
| $c_{n,P}$ | Phosphorous content in plant dry weight | $g kg DW^{-1}$ |
| $c_{n,K}$ | Potassium content in plant dry weight | $g kg DW^{-1}$ |
| $c_{n,Ca}$ | Calcium content in plant dry weight | $g kg DW^{-1}$ |
| $c_{\tau}$ | Root to total dry weight ratio | Adim. |
| **Plant model calibration** | | |
| $A_{1}$ | Area assumed in the experiments of Ford and Thorne ^1^ | $m^{2}$ |
| $A_{2}$ | Area assumed in the experiments of Chowdhury et al.^2^ | $m^{2}$ |
| $f_{er}$ | Error function | $kg DW m^{-2}$ |
| $x_{i}$ | Dry weight per unit area reported by the articles for experiment $i$ | $kg DW m^{-2}$ |
| $\hat{x}_{i}$ | Dry weight per unit area reported by the articles for experiment $i$ | $kg DW m^{-2}$ |
| **Mass and energy balances** | | |
| $\dot{m}_{l}$ | Air leakage rate out of the IVF | $\mathrm{kg}s^{-1}$ |
| $\dot{m}_{l,ACH}$ | Air leakage rate out of the IVF through the cracks | $\mathrm{kg}s^{-1}$ |
| $\dot{m}_{l,d}$ | Air leakage rate out of the IVF through the door | $\mathrm{kg}s^{-1}$ |
| $\Delta p$ | Pressure difference across the IVF door | $\mathrm{Pa}$ |
| $C_{A}$ | Airflow coefficient | $cfm ft^{-2} in_{H_{2}O}^{-0.5}$ |
| $A_{d}$ | Area of the IVF door | $m^{2}$ |
| $\rho_{air}$ | Air density | $\mathrm{kg}m^{-3}$ |
| $N$ | Number of people crossing the IVF per hour | $h^{-1}$ |
| $V_{IVF}$ | Air volume inside the IVF | $m^{3}$ |
| $\Delta t$ | Time step | $s$ |
| $\dot{m}_{f}$ | Mass flowrate of air supplied to the IVF | $\mathrm{kg}s^{-1}$ |
| $\dot{m}_{CO_{2},f}$ | Mass flowrate of CO_2_ in the air supplied to the IVF | $\mathrm{kg}s^{-1}$ |
| $\dot{m}_{CO_{2},s}$ | Mass flowrate of CO_2_ supplied by the cylinders | $\mathrm{kg}s^{-1}$ |
| $\dot{m}_{CO_{2},l}$ | Mass flowrate of CO_2_ leaking out the IVF | $\mathrm{kg}s^{-1}$ |
| $\dot{m}_{CO_{2},ppl}$ | Mass flowrate of CO_2_ supplied by the human workers | $\mathrm{kg}s^{-1}$ |
| $\dot{m}_{CO_{2},plt}$ | Mass flowrate of CO_2_ traded with the plants | $\mathrm{kg}s^{-1}$ |
| $\Delta\dot{m}_{CO_{2}}$ | Mass flowrate of CO_2_ imbalance | $\mathrm{kg}s^{-1}$ |
| $\dot{m}_{w,f}$ | Mass flowrate of moisture in the air supplied to the IVF | $\mathrm{kg}s^{-1}$ |
| $\dot{m}_{w,l}$ | Mass flowrate of moisture leaking out the IVF | $\mathrm{kg}s^{-1}$ |
| $\dot{m}_{w,s}$ | Mass flowrate of moisture supplied by the humidifiers | $\mathrm{kg}s^{-1}$ |
| $\dot{m}_{w,cond}$ | Mass flowrate of moisture condensed by the air handling unit (AHU) | $\mathrm{kg}s^{-1}$ |
| $\dot{m}_{w,ppl}$ | Mass flowrate of moisture supplied by the human workers | $\mathrm{kg}s^{-1}$ |
| $\dot{m}_{w,plt,t}$ | Mass flowrate of moisture released by the plants | $\mathrm{kg}s^{-1}$ |
| $q_{f}$ | Heat in the air supplied to the IVF | $W$ |
| $q_{l}$ | Heat leaking out the IVF through air leakages | $W$ |
| $q_{h}$ | Heat supplied by the heating resistance at the AHU | $W$ |
| $q_{c}$ | Heat removed by the cooling heat pump at the AHU | $W$ |
| $q_{walls}$ | Heat traded with the basement through the walls | $W$ |
| $q_{ppl}$ | Heat supplied by the human workers | $W$ |
| $q_{plt}$ | Heat absorbed by the plants | $W$ |
| $q_{equip}$ | Heat supplied by the IVF equipment | $W$ |
| $c_{p,l}$ | Specific heat at constant pressure of liquid water | $J kg^{-1} K^{-1}$ |
| $c_{p,v}$ | Specific heat at constant pressure of water vapor | $J kg^{-1} K^{-1}$ |
| $c_{p,CO_{2}}$ | Specific heat at constant pressure of CO_2_ | $J kg^{-1} K^{-1}$ |
| $c_{p,air}$ | Specific heat at constant pressure of air | $J kg^{-1} K^{-1}$ |
| $d_{cl}$ | Thickness of cladding layer in the IVF walls | $m$ |
| $d_{f}$ | Thickness of foam layer in the IVF walls | $m$ |
| $k_{cl}$ | Conductivity of cladding layer in the IVF walls | $W m^{-1} K^{-1}$ |
| $k_{f}$ | Conductivity of foam layer in the IVF walls | $W m^{-1} K^{-1}$ |
| $h_{air}$ | Heat transfer coefficient of the air surrounding the IVF walls | $W m^{-2} K^{-1}$ |
| $A_{wall,i}$ | Area of the wall $i$ of the IVF | $m^{2}$ |
| $h_{fg}$ | Specific enthalpy of vaporization of water | $J kg^{-1}$ |
| $T_{cond}$ | Temperature at the condenser of the AHU | $℃$ |
| $E_{AHU}$ | Electricity use of the AHU | $W$ |
| $COP_{h}$ | Coefficient of performance of the heating resistance at the AHU | Adim. |
| $COP_{c}$ | Coefficient of performance of the cooling heat pump at the AHU | Adim. |
| **Life cycle inventories** | | |
| $n_{Ref}$ | Number of CO_2_ cylinder refills necessary per week | Items |
| $T_{car}$ | Transportation by light vehicle | $kg km$ |
| $T_{trk}$ | Transportation by 16-ton freight truck | $kg km$ |
| $n_{GH}$ | Number of IVF infrastructures required | Items |
| $n_{LEDs}$ | Number of LED systems required | Items |
| $n_{cyl}$ | Number of aluminum cylinders required | Items |
| $n_{f,c}$ | Number of flowrate controllers required | Items |
| $n_{h}$ | Number of humidifiers required | Items |
| $n_{tr}$ | Number of sets of treatment equipment required | Items |
| $m_{w,T}$ | Mass of total water required | $\mathrm{kg}$ |
| $m_{CO_{2},s}$ | Mass of CO*2* supplied to the IVF | $\mathrm{kg}$ |
| $m_{Ca\left( NO_{3} \right)_{2}}$ | Mass of calcium nitrate supplied to the plants | $\mathrm{kg}$ |
| $m_{NH_{4}H_{2}PO_{4}}$ | Mass of monoammonium phosphate supplied to the plants | $\mathrm{kg}$ |
| $m_{KNO_{3}}$ | Mass of potassium nitrate supplied to the plants | $\mathrm{kg}$ |
| $E^{T}$ | Electricity required | $\mathrm{kWh}$ |
| $m_{FW,T}$ | Mass of total fresh weight produced | $\mathrm{kg}$ |
| $m_{CO_{2},e}$ | Mass of CO_2_ leaked to the atmosphere | $\mathrm{kg}$ |
| $m_{FW,T,s}$ | Mass of shoot fresh weight harvested | $\mathrm{kg}$ |
| $m_{FW,T,r}$ | Mass of root fresh weight composted | $\mathrm{kg}$ |
| $m_{sub}$ | Mass of substrate composted | $\mathrm{kg}$ |

S.3. References

1. Ford, M. A. & Thorne, G. N. Effect of CO2 Concentration on Growth of Sugar-beet, Barley, Kale, and Maize. *Ann Bot* **31**, 629–644 (1967).

2. Chowdhury, M. *et al.* Effects of temperature, relative humidity, and carbon dioxide concentration on growth and glucosinolate content of kale grown in a plant factory. *Foods* **10**, 1524 (2021).

3. Parkes, M. G., Tovar, J. P. C., Dourado, F., Domingos, T. & Teixeira, R. F. M. Life Cycle Assessment of a prospective technology for building-integrated production of broccoli microgreens. *Atmosphere (Basel)* **13**, 1317 (2022).

4. van Henten, E. Greenhouse climate management: an optimal control approach. (1994).

5. van Henten, E. J. Validation of a dynamic lettuce growth model for greenhouse climate control. *Agric Syst* **45**, 55–72 (1994).

6. Carnivero. Light Sources - Carnivero. *Carnivero, Carnivorous Plants and Tropicals Nursery* https://www.carnivero.com/pages/light-sources (2022).

7. Goudriaan, J. & Monteith, J. L. A Mathematical Function for Crop Growth Based on Light Interception and Leaf Area Expansion. *Ann Bot* **66**, 695–701 (1990).

8. Waterland, N. L., Moon, Y., Tou, J. C. & Kopsell, D. A. Differences in Leaf Color and Stage of Development at Harvest Influenced Phytochemical Content in Three Cultivars of Kale (Brassica oleracea L. and B. napus. *Journal of Agricultural Science* **11**, 14–21 (2019).

9. Waterland, N. L. *et al.* Mineral Content Differs among Microgreen, Baby Leaf, and Adult Stages in Three Cultivars of Kale. *HortScience* **52**, 566–571 (2017).

10. Li, T., Lalk, G. T., Arthur, J. D., Johnson, M. H. & Bi, G. Shoot production and mineral nutrients of five microgreens as affected by hydroponic substrate type and post-emergent fertilization. *Horticulturae* **7**, 129 (2021).

11. Kozai, T. & Niu, G. Chapter 8. in *Plant Factory: An Indoor Vertical Farming System for Efficient Quality Food Production* (eds. Kozai, T., Niu, G. & Takagaki, M.) 129–140 (Academic Press, 2016). doi:10.1016/C2014-0-01039-8.

12. Cho, H., Gowri, K. & Liu, B. *Energy Saving Impact of ASHRAE 90.1 Vestibule Requirements: Modeling of Air Infiltration through Door Openings*. https://www.pnnl.gov/main/publications/external/technical_reports/PNNL-20026.pdf (2010).

13. HPCi Media Limited. Cleanroom tightness and room pressurisation: Discrepancy? *Cleanroom technology* https://www.cleanroomtechnology.com/news/article_page/Cleanroom_tightness_and_room_pressurisation_Discrepancy/169785 (2020).

14. Kozai, T. & Niu, G. Chapter 4. in *Plant Factory: An Indoor Vertical Farming System for Efficient Quality Food Production* (eds. Kozai, T., Niu, G. & Takagaki, M.) 69–90 (Academic Press, 2016). doi:10.1016/C2014-0-01039-8.

15. Mehnert, P., Bröde, P. & Griefahn, B. Gender-related difference in sweat loss and its impact on exposure limits to heat stress. *Int J Ind Ergon* **29**, 343–351 (2001).

16. Zieliński, J. & Przybylski, J. How much water is lost during breathing? *Adv Respir Med* **80**, 339–342 (2012).

17. The Engineering Toolbox. The Engineering ToolBox. https://www.engineeringtoolbox.com/ (2011).

18. Haskell, W. L. *et al.* Physical activity and public health: updated recommendation for adults from the American College of Sports Medicine and the American Heart Association. *Circulation* **116**, 1081–1093 (2007).

19. Carvill, J. Chapter 3. in *Mechanical Engineer’s Data Handbook* (ed. Carvill, J.) 102–145 (1993). doi:10.1016/B978-0-08-051135-1.50008-X.

20. Zhang, H., Fang, W.-Z., Li, Y.-M. & Tao, W.-Q. Experimental study of the thermal conductivity of polyurethane foams. *Appl Therm Eng* **115**, 528–538 (2017).

21. Incropera, F. P., DeWitt, D. P., Bergman, T. L. & Lavine, A. S. *Fundamentals of Heat and Mass Transfer*. (John Wiley & Sons, 2006).

22. ISMA. Electroválvulas normalmente fechadas para gás - Série MD11-12-13 | ISMA - Equipamentos para condução, controlo e gestão de fluidos. https://www.isma.pt/pt/produtos/electrovalvulas-e-bombas-de-solenoide/filtros-e-electrovalvulas/electrovalvulas-para-gas-normalmente-fechadas-serie-md11-12-13/ (2011).

23. CAREL. humiDisk: humidificador centrífugo/ centrifugal humidifier. vol. 5 https://www.carel.com/documents/10191/0/+030222022/12227bf0-1a16-48eb-a806-2f47cbe9b3a8?version=1.7 (2021).

24. The Engineering Toolbox. Electric Motors - Efficiency. *2010* vol. 10 https://www.engineeringtoolbox.com/electrical-motor-efficiency-d_655.html.

25. Beijer Ref. Axial fans. Main catalogue. 2016 Edition. *Beijer - UAB ‘Beijer Ref Lithuania’* 178–179 https://www.refrigeration.lt/uploads/Products/product_18424/Ziehl-Abegg_axial_fans_main_catalogue_2016.pdf (2018).

26. Ministério das Obras Públicas, T. e C. *Decreto-Lei n.^o^ 80/2006, de 4 de abril*. *Regulamento das Características de Comportamento Térmico dos Edifícios, Artigo 20^o^* (2006).

27. APREN. APREN - Production. https://www.apren.pt/en/renewable-energies/production (2022).

28. Luxfer Gas Cylinders. Aluminum Cylinders | Luxfer Gas Cylinders. vol. 14 https://www.luxfercylinders.com/products/agriculture/l6x-aluminum-cylinder-beverage (2020).

29. European Chemical Site Promotion Platform. Chemmed Cluster Tarragona - Chemical park - Spain. vol. 15 https://chemicalparks.eu/parks/chemmed-cluster-tarragona (2018).

30. Messer. Distribuidores - MesserGas Distribuição de Gases Industriais Lda. https://www.messer.pt/distribuidores (2022).

31. Messer. Environmental & climate protection. https://corporate.messergroup.com/environmental-and-climate-protection (2020).

32. Advameg Inc. How oxygen tank is made - material, making, history, used, product, machine, History, Raw Materials, Design. http://www.madehow.com/Volume-7/Oxygen-Tank.html (2006).

33. Scharf, S., Bergedieck, N., Riedel, E., Richter, H. & Stein, N. Unlocking Sustainability Potentials in Heat Treatment Processes. *Sustainability* **12**, 6457 (2020).

34. Gas Cylinder Source. CO2 Carbon Dioxide Valve - CGA320 - 1.125" - 12 UNF - Cavagna | Gas Cylinder Source. https://gascylindersource.com/shop/co2-carbon-dioxide-valves/co2-carbon-dioxide-valve-cga320-1-125-12-unf-cavagna/ (2021).

35. Holding, X. Brass valve hot forging machine. *Youtube* https://youtu.be/SW-I0lzWU40 (2018).

36. Cyl-Tec, I. Aluminum CO2 Cylinders » Cyl-Tec, Inc. https://cyl-tec.com/product/aluminum-co2-cylinders/ (2021).

37. Aralab. Fitoclima 48000 PLH - Mod. MI377. Preprint at (2021).

38. CAREL. UC humiDisk 65 - UC humiDisk - Humidifiers. *Carel Humidification Parts & Parametric Controls* https://www.carelparts.com/humidifiers/uc-humidisk/uc-humidisk-10-2.html (2019).

39. CAREL. UC humiDisk 10 - UC humiDisk - Humidifiers. *Carel Humidification Parts & Parametric Controls* https://www.carelparts.com/humidifiers/uc-humidisk/uc-humidisk-10.html (2019).

40. CAREL. CAREL announces a new plant in Croatia. https://www.carel.com/news-detail/-/asset_publisher/FWCUXAoY43Lv/content/carel-announces-a-new-plant-in-croatia/10191 (2021).

41. CAREL. Portugal. https://www.carel.com/distributors-portugal (2018).
